# Discovering the RNA-Binding Proteome of Plant Leaves with an Improved RNA Interactome Capture Method

**DOI:** 10.3390/biom10040661

**Published:** 2020-04-24

**Authors:** Marcel Bach-Pages, Felix Homma, Jiorgos Kourelis, Farnusch Kaschani, Shabaz Mohammed, Markus Kaiser, Renier A. L. van der Hoorn, Alfredo Castello, Gail M. Preston

**Affiliations:** 1 Department of Plant Sciences, University of Oxford, South Parks Road, Oxford OX1 3RB, UK; marcel.bachpages@plants.ox.ac.uk (M.B.-P.); felix.homma@bnc.ox.ac.uk (F.H.); Jiorgos.Kourelis@tsl.ac.uk (J.K.); renier.vanderhoorn@plants.ox.ac.uk (R.A.L.v.d.H.); 2 Fakultät für Biologie, Universität Duisburg-Essen, North Rhine-Westphalia, 45117 Essen, Germany; farnusch.kaschani@uni-due.de (F.K.); markus.kaiser@uni-due.de (M.K.); 3 Department of Biochemistry, University of Oxford, South Parks Road, Oxford OX1 3QU, UK; shabaz.mohammed@chem.ox.ac.uk

**Keywords:** RNA-binding proteins, RBP, protein–RNA interaction, RNA-binding proteome, RBPome, RNA interactome capture, RIC, ptRIC, Arabidopsis, plant

## Abstract

RNA-binding proteins (RBPs) play a crucial role in regulating RNA function and fate. However, the full complement of RBPs has only recently begun to be uncovered through proteome-wide approaches such as RNA interactome capture (RIC). RIC has been applied to various cell lines and organisms, including plants, greatly expanding the repertoire of RBPs. However, several technical challenges have limited the efficacy of RIC when applied to plant tissues. Here, we report an improved version of RIC that overcomes the difficulties imposed by leaf tissue. Using this improved RIC method in Arabidopsis leaves, we identified 717 RBPs, generating a deep RNA-binding proteome for leaf tissues. While 75% of these RBPs can be linked to RNA biology, the remaining 25% were previously not known to interact with RNA. Interestingly, we observed that a large number of proteins related to photosynthesis associate with RNA in vivo, including proteins from the four major photosynthetic supercomplexes. As has previously been reported for mammals, a large proportion of leaf RBPs lack known RNA-binding domains, suggesting unconventional modes of RNA binding. We anticipate that this improved RIC method will provide critical insights into RNA metabolism in plants, including how cellular RBPs respond to environmental, physiological and pathological cues.

## 1. Introduction

RNA-binding proteins (RBPs) interact with RNAs to form dynamic ribonucleoprotein (RNP) complexes that regulate the fate and function of RNA at virtually every step of its life cycle [1]. Therefore, RBPs are key players in the control of gene expression by regulating the synthesis, processing (capping, splicing and polyadenylation), editing, transport, storage, surveillance/quality control, function, translation and turnover of RNA [1]. Because RBPs have a critical role in cell biology, the complement of RBPs, referred to here as the RNA-binding proteome (RBPome), is tightly regulated and remodelled in response to alterations in environmental conditions and variations in cellular states [2,3,4,5]. Hence, elucidating the composition of the RBPome becomes crucial to understand RNA biology in different tissues and experimental conditions.

In the last decade, many efforts have been undertaken to identify RBPs in a comprehensive manner. For example, in vitro studies have employed protein arrays to determine proteins that capture fluorescently-labelled RNAs [6,7], or immobilised RNAs as baits to isolate RBPs [8]. These studies identified hundreds of proteins with the capacity to interact with RNA in vitro; however, whether these proteins interact with RNA in a physiological, native environment remained unknown [9]. This is because in vitro studies can detect protein–RNA interactions that may not occur in a cellular context, since RNA is a highly electronegative molecule that can interact with positively charged proteins non-specifically under non-physiological conditions. Moreover, certain protein and RNA molecules may not interact in vivo because they are present in different subcellular localisations, or because the positively charged surface of the protein instead mediates protein–protein or protein–DNA interactions in the cell. Many in silico algorithms have been developed to identify novel RBPs by searching for domains with homology to well-established RNA-binding domains (RBDs). This has helped to classify hundreds of proteins as putative RBPs in different organisms [10]. However, these computational approaches are based on homology and thus cannot discover RBPs with unconventional architectures or sequences, or intrinsically disordered regions (IDRs) with in vivo RNA-binding capacity [11,12].

A recently developed approach termed RNA interactome capture (RIC) can systematically and comprehensively identify the proteins that interact with polyadenylated RNAs in living cells [9,13,14]. RIC employs ultraviolet (UV) irradiation of cells to promote RNA-to-protein crosslinking, followed by capture of poly(A) RNAs with oligo(dT) under denaturing conditions. Proteins crosslinked to isolated poly(A) RNA are identified by quantitative mass spectrometry. RIC has multiple advantages over previous techniques used to isolate RBPs: (i) RIC allows the identification of proteins directly associated with RNA. Since UV light does not promote protein–protein crosslinking, RIC will not efficiently identify proteins interacting with RNA indirectly [15]; (ii) RIC uncovers RBPs acting in their native environment, as UV crosslinking is applied to living cells; (iii) RIC unbiasedly identifies both canonical and unconventional RBPs; (iv) RIC can be applied to comparative studies that aim to uncover RBP dynamics in response to experimental changes, as it can be coupled to quantitative proteomics [2,3,4]. On the other hand, RIC will not identify a given RBP when (i) the RBP does not interact with poly(A) RNA; (ii) the RBP is not expressed or active in the model system under study or under the experimental conditions used; (iii) the RBP does not efficiently UV-crosslink to RNA due to the geometry between nucleotides and amino acids, as RIC favours the crosslinking of proteins that interact with the nucleotide bases over the phosphate backbone [12]; and (iv) as with any other proteomic approach, peptide abundance and amino acid sequence may influence the ability of RIC to identify RBPs.

Since 2012, RIC has been applied to multiple organisms including Trypanosoma brucei [16], Leishmania [17,18], Plasmodium falciparum [19], Saccharomyces cerevisiae [20,21,22,23], Caenorhabditis elegans [23], Drosophila melanogaster [2,24], Danio rerio [25], Mus musculus [26,27,28,29] and different Homo sapiens cell lines [3,12,13,14,21,30,31]. This has greatly expanded the repertoire of RBPs, and with it our knowledge of RNA biology [32]. RIC has also been applied to different tissues of the model plant Arabidopsis thaliana [33]: etiolated seedlings [34], cell cultures [35,36] and protoplasts derived from the mesophyll [37,38]. Although a recent work applied RIC to plant leaves [35], this study only identified 27 leaf RBPs, which is a very small fraction of the expected plant RBPome. Indeed, the RBPomes generated in the other plant systems contain ~226–372 RBPs [32]. Hence, the RBPome of leaves and other physiologically relevant plant tissues still needs to be uncovered comprehensively. Applying RIC to plant leaves is challenging because UV-crosslinking efficiency can be reduced due to the thickness of leaves and the presence of UV-absorbing pigments such as chlorophyll [39]. Additionally, the composition of leaves is more complex than human cell lines due to the presence of the cell wall and additional secondary metabolites [39].

In this study, we report an improved RIC protocol that allows the efficient application of RIC to plant leaves, which are broadly used to study plant biology and are critical for understanding plant physiology and functioning. Using this modified protocol, referred to here as ‘plant RNA interactome capture’ (ptRIC), we identified a comprehensive leaf RBPome for the model plant *Arabidopsis thaliana*, comprising 717 RBPs. While 75% of the leaf RBPs were annotated to bind RNA, 25% of the RBPs were previously not known to interact with RNA, including metabolic enzymes and proteins from the photosynthetic apparatus. Our analysis revealed cellular RBPs harbouring classic RBDs as well as dozens of proteins lacking known RBDs, indicating unconventional modes of protein–RNA association. ptRIC offers an unprecedented opportunity to study the dynamics of RBPs under different developmental and cellular stages, and in response to changes in environmental conditions. These analyses will allow us to integrate RBP activity into multi-omic analyses to understand plant gene expression.

## 2. Materials and Methods

### 2.1. Plant Material

Mature *Arabidopsis thaliana* plants (5–6 weeks old) of the Col-0 ecotype were used for all experiments. Plants were grown in soil at neutral day conditions (12 h light, 12 h dark) at 20 °C and a light intensity of approximately 100 μmol/m^2^/s.

### 2.2. Plant RNA Interactome Capture (ptRIC)

A step-by-step protocol including all information about the reagents used is included in the Supplementary Materials.

#### 2.2.1. UV Crosslinking

For UV crosslinking, leaves of mature Arabidopsis plants were excised and placed on a plastic sheet on top of ice pads to prevent sample overheating. The leaves were crosslinked three times with 150 mJ/cm^2^ of UV light at 254 nm wavelength. We allowed 30 s pause in between irradiations. For the non-crosslinked (NoCL) negative control, leaves were placed on ice for approximately the same time the crosslinked (CL) samples were maintained on the ice pads during irradiation (~3 min). After irradiation, the leaves were immediately frozen in liquid nitrogen to preserve the molecular interactions and sample integrity. Both CL and NoCL samples were processed in parallel following the same protocol.

#### 2.2.2. Cell Lysis

Leaf tissue was ground to a fine powder in liquid nitrogen using a mortar and pestle. Next, 1.2 g of tissue was mixed with 12 mL of lysis buffer in a 50 mL tube. The lysates were kept on ice to minimise RNA degradation. To further homogenise the lysates, a Potter–Elvehjem homogeniser (Merck, Darmstadt, Germany) was used. Samples were homogenised for 1 min, while they were kept on ice to avoid sample overheating. The lysates were cleared by centrifugation (4000 rpm, 10 min, 4 °C) and filtration of the supernatant through Miracloth (Merck, Darmstadt, Germany). To shear the gDNA, lysates were passed through a narrow needle (27 G) five times. The lysates were cleared again by centrifugation (4000 rpm, 10 min, 4 °C) and filtration of the supernatant through Miracloth.

#### 2.2.3. Oligo(dT) Capture

Before starting the oligo(dT) capture, aliquots of 200–500 μL of the input samples (whole cell lysates/total proteomes) were taken and stored at −80 °C. Oligo(dT) beads (250 μL/sample) were activated by washing them three times with lysis buffer (20 mM Tris-HCl (pH 7.5), 500 mM LiCl, 1 mM EDTA, 0.5% LiDS (*wt*/*v*), 0.02% IGEPAL,2.5% PVP40 (*wt*/*v*), 1% B-ME (*v*/*v*), 5mM DTT, protease inhibitor and RNase inhibitor), using a magnet to trap the magnetic beads followed by supernatant removal. Next, 250 μL of beads was added to each of the lysates and incubated at 4 °C for 1 h in a rotator (10 rpm) to allow hybridisation of the oligo(dT) to the poly(A) tail of the RNAs. The beads were captured on a magnet for ~20–30 min at 4 °C, making sure the supernatant was cleared. The supernatant was collected and stored on ice for a second round of capture. The beads were then washed with 1.5 mL of different buffers with an incubation of 5 min on ice, inverting the tube every 30–60 s, followed by magnet capture and supernatant removal. First, the beads were washed with lysis buffer, which is a stringent buffer that allows the removal of unspecific binders sticking to the oligo(dT) beads or bound to the RNAs non-covalently or via protein–protein interactions. The beads were then washed with harsh buffer (20 mM Tris-HCl (pH 7.5), 2 M LiCl, 1 mM EDTA, 1% LiDS (*wt*/*v*), 0.02% IGEPAL (*v*/*v*), 5 mM DTT) at room temperature to increase the removal of unspecific binders and avoid precipitation of the LiDS. The harsh buffer contained higher concentrations of the ionic detergent LiDS and LiCL to further increase the stringency of the capture. The beads were then washed two times with buffer I (20 mM Tris-HCl (pH 7.5), 500 mM LiCl, 1 mM EDTA, 0.1% LiDS (*wt*/*v*), 0.02% IGEPAL (*v*/*v*), 5 mM DTT), two times with buffer II (20 mM Tris-HCl (pH 7.5), 500 mM LiCl, 1 mM EDTA, 0.02% IGEPAL (*v*/*v*), 5 mM DTT), one time with buffer III with detergent (20 mM Tris-HCl (pH 7.5), 200 mM LiCl, 1 mM EDTA, 0.02% IGEPAL (*v*/*v*), 5 mM DTT), and one time with buffer III without detergent (20 mM Tris-HCl (pH 7.5), 200 mM LiCl, 1 mM EDTA, 5 mM DTT), as this detergent interferes with downstream mass spectrometry analyses. To elute the RNA–protein complexes, beads were resuspended and incubated with 300 μL of elution buffer (20 mM Tris-HCl (pH 7.5), 1 mM EDTA) for 3 min at 55 °C. The beads were pelleted using a magnet and the supernatant containing the RNA–protein complexes was transferred to a new tube and stored at −80 °C.

For the second round of capture, the beads were washed three times with lysis buffer and added to the supernatants that were recovered from the first capture and kept at 4 °C. Beads were only reused for the same condition (i.e., the beads of Treatment 1 were reused for the second round of capture of samples of Treatment 1).

### 2.3. Downstream Applications of Isolated RNA–Protein Complexes

#### 2.3.1. RNA Quantification and Normalization

For each of the samples, the two eluates from different rounds of capture were pooled and quantified using a NanoDrop spectrophotometer (Thermo Fisher Scientific, Waltham, MA, USA) Elution buffer was used to adjust the volume of the samples so that the same amount of RNA was present in each sample.

#### 2.3.2. RNase Digestion

After isolation of the RNA–protein complexes, we treated each of the eluates with 4 μL of RNase A and T1 mix (RNase A and RNase T1 mixed at equal proportions and diluted 1/100) for 1 h at 37 °C followed by incubation for 15 min at 50 °C. Samples were then analysed as follows: (i) Western blotting with specific antibodies; (ii) silver staining for total protein analysis; and (iii) quantitative mass spectrometry to identify and quantify the isolated proteins. Out of the 600 μL of the eluate samples, 50 μL was used for Western blotting and silver staining analyses, and the remaining material (550 μL) was used for mass spectrometry (stored at −80 °C).

#### 2.3.3. Protein Concentration and Western Blot or Silver Staining

For Western blot or silver staining analyses, proteins were concentrated by centrifugation using an Amicon centrifugal filter (Merck, Darmstadt, Germany) of 3 KDa cut-off (15,000 rpm, 4 °C, 1–2 h). The volume recovered from each filter was measured and normalised using elution buffer to proceed with the same volume in each sample. The inputs (whole cell lysates/total proteome) and the eluates (RBPs) were mixed with protein loading buffer (1/4, *v*/*v*) and incubated for 4 min at 95 °C. Proteins were separated in a 10–12% acrylamide gel followed by western blot or silver staining. Western blots were performed following standard procedures [40] using the following antibodies: anti-RH3 (Agrisera, Vännäs, Sweden, AS132714), anti-HSC70 (Agrisera, AS08371), anti-H3 (Abcam, Cambridge, UK ab1791) and anti-UGPase (Agrisera, AS05086). Silver staining was performed using SilverQuest (Invitrogen, cat. no. LC6070) following the manufacturer’s instructions. Samples were prepared and analysed by mass spectrometry, as explained in the Supplementary Materials and Methods.

### 2.4. Data Analysis

#### Statistical Analysis of the RBPome

All statistical analyses were conducted in R [41]. Contaminant protein groups, including reversed sequences and protein groups identified by site, were filtered from our dataset. Raw intensities were used when comparing NoCL and CL samples because label-free quantification (LFQ) intensities are normalised and thus are not suitable for experiments in which one condition is rich in proteins (CL) while the other is devoid of proteins (NoCL; Figure S1A) [42]. The proteins from the CL samples were only considered for analysis if quantified in at least three out of four replicates. Some of the identified peptides were not unique and could not be assigned to a single protein. For these, we conducted analyses using the protein groups, although we could not confidently assign the peptide to one unique protein.

The identified proteins were separated into two groups and analysed using different methods as follows. The proteins with values in the CL samples, which were not detected in any of the NoCL samples (negative control) were analysed using a modified version of the semi-quantitative approach described by Sysoev and colleagues [2]. We classified as ‘RBPs’ proteins quantified in at least three out of four replicates in the CL replicates, but detected in none of the NoCL replicates. For proteins that had intensity values in both CL and NoCL, we used a quantitative method. Briefly, the missing values were imputed using the impute.minDet function [41] and the ratios of CL/NoCL were calculated. Statistical analysis of CL/NoCL enrichment samples was performed using a moderate *t*-test implemented in the R/Bioconductor package limma [43]. The resulting *p*-values were corrected for multiple testing using Benjamini–Hochberg [44] to calculate the false discovery rate (FDR; *p*-adjusted). Proteins with a log_2_ fold change [CL/NoCL] > 1.5 and an adjusted *p*-value (FDR) < 0.01 were defined as ‘RBPs’, whereas the remaining proteins (‘non-enriched in +UV/−UV’) were discarded to avoid a high incidence of false positives. During the process of optimising ptRIC, we analysed a pilot experiment by mass spectrometry. For this early experiment, we analysed one CL and one NoCL sample. Therefore, in this pilot experiment we classified a protein as RBP if log_2_FC [CL/NoCL] ≥ 2.

The proteins defined as RBPs by either semi-quantitative or quantitative methods were pooled to define a high-confidence Arabidopsis leaf RBPome. Bioinformatic analyses of the Arabidopsis leaf RBPome were performed as described in the Supplementary Materials and Methods.

### 2.5. Data Availability

The mass spectrometry proteomics data were deposited into the ProteomeXchange Consortium via the PRIDE [45] partner repository (https://www.ebi.ac.uk/pride/archive/) with the dataset identifier PXD018141.

## 3. Results and Discussion

### 3.1. Adapting RIC to Plant Leaves

**Figure 1:**
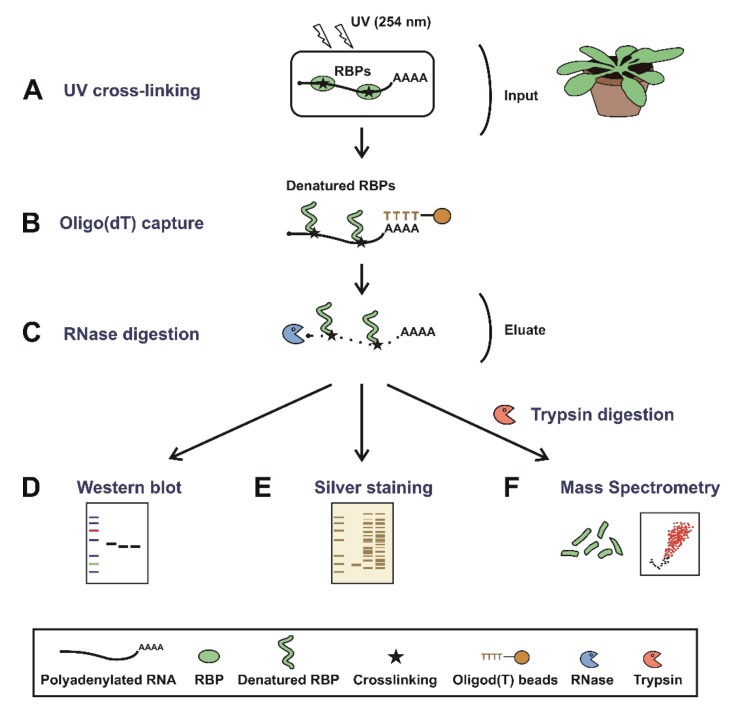
Schematic representation of plant RNA interactome capture (ptRIC). Mature plants (leaves) are irradiated with UV light at 254 nm to promote crosslinking between RNAs and proteins that are in intimate contact (**A**). Next, cells are lysed and polyadenylated RNAs pulled down using oligo(dT) magnetic beads (**B**). After stringent washes, the RNA–protein complexes are recovered and the RNA-binding proteins (RBPs) released by RNase digestion (**C**). The proteins can be quantitatively analysed by (**D**) Western blot, (**E**) silver staining or (**F**) mass spectrometry after trypsin digestion.

**Figure 2:**
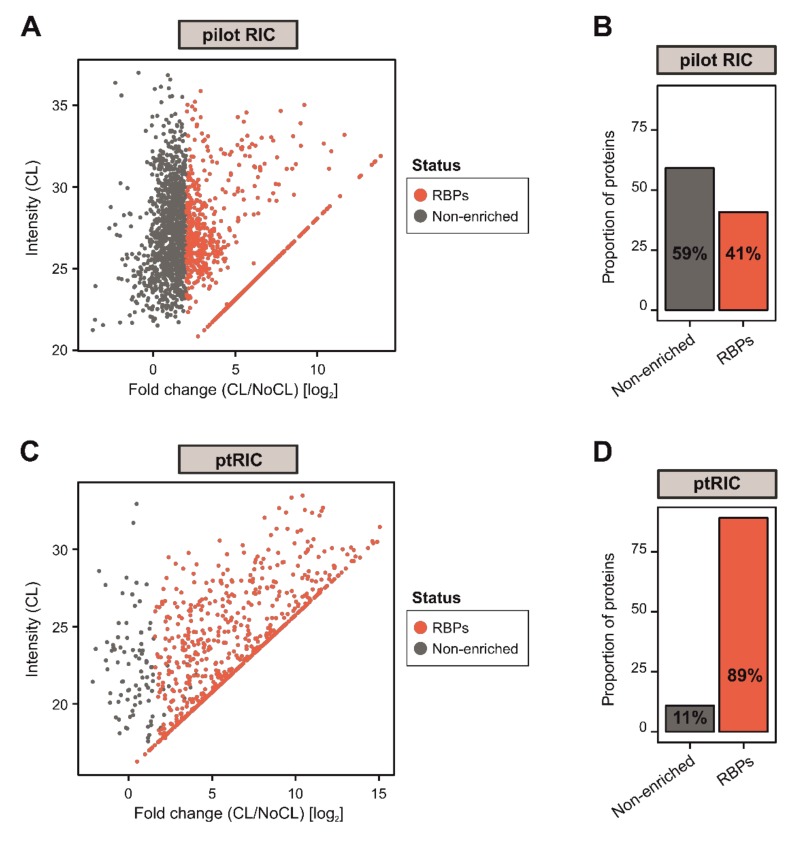
Development of plant RNA interactome capture (ptRIC), an improved RIC protocol to isolate RNA-binding proteins (RBPs) actively bound to RNA from leaf tissue. Scatter plots depict the log_2_ fold change between no UV crosslinking (NoCL) and UV crosslinking (CL) treatments (log_2_ FC(CL/NoCL)) and the signal intensity in the CL sample for each protein (dots) using data from four biological replicates from the pilot RIC (**A**) or ptRIC (**C**). The colour of the dots indicates whether proteins were classified as ‘RBPs’ (red) or +UV/−UV ‘non-enriched proteins’ (grey). Bar charts show the proportion of proteins classified as ‘RBPs’ or +UV/−UV ‘non-enriched’ in the pilot RIC (**B**) and the ptRIC (**D**) experiments.

To comprehensively uncover the complement of cellular RBPs that participate in plant physiology, we sought to further improve RIC to function efficiently in leaves. In our initial attempt, we applied the RIC settings described by Reichel and colleagues [34] to mature leaves of *Arabidopsis thaliana*. In brief, leaves were detached from the plants and irradiated three times with UV-C light at 150 mJ/cm^2^ (CL) to induce covalent bonds between RNAs and proteins that were in intimate contact (≤2Å). Next, 1.5 g of tissue was disrupted and cells were lysed, followed by capture of poly(A) RNAs and covalently linked proteins using oligo(dT) magnetic beads. This isolation was performed under stringent denaturing conditions using ionic detergents and high LiCl concentrations to exclusively isolate direct RNA binders, as described by Reichel [34]. The RNAs were subsequently degraded using RNases and the proteins associated with RNAs were analysed by silver staining and mass spectrometry (Figure 1). A sample omitting the UV irradiation (NoCL) was processed in parallel as a negative control. Although proteomic analysis revealed nearly two thousand proteins, only 41% of these were categorised as RBPs (log_2_FC [CL/NoCL] ≥ 2). More importantly, 59% were not enriched over the non−UV irradiated negative control, representing a set of unspecific binders that co-purified with RNA in a UV-independent manner (Figure 2A,B). These unspecific binders resulted in a higher sample complexity and increased the chance for false positives in the determination of the RBPome.

To circumvent this problem, we optimised multiple parameters that we suspected were affecting RBP recovery. A sufficient UV dose is critical for efficient protein–RNA crosslinking; however, excessive UV exposure can lead to RNA damage and, therefore, reduction of RBP yield [20,46]. Leaf tissue is highly refractory to UV light due to its thickness and the presence of pigments able to absorb UV light [39]. Hence, we reasoned that the processing of the tissue and the UV dose could be important factors determining UV-dependent protein recovery. We tested UV irradiation of either full rosettes, intact or pulverised leaves and determined that intact detached leaves were the most optimal starting material (Figure S2A). We then assessed different UV doses and regimens (from 150 mJ/cm^2^ to triple irradiations with 800 mJ/cm^2^) and determined that the optimal crosslinking protocol was three irradiations at 150 mJ/cm^2^ spaced by a 30 s pause, as previously proposed by Reichel and colleagues [34]. Moreover, we irradiated both adaxial and abaxial sides of the leaves to maximise uniformity in the crosslinking throughout the leaf.

We suspected that some of the non-enriched unspecific binders (Figure 2A,B) could be associated with cellular debris trapped in the beads, resulting from incomplete homogenization of the tissue. To solve this problem, we increased the lysis-buffer-to-tissue ratio and added an additional step of homogenisation with a Potter–Elvehjem homogeniser. DNA-binding proteins can also crosslink with DNA upon UV exposure, so we passed the lysates through a narrow needle to shear any genomic DNA that could stick non-specifically to the beads and increase protein background. Furthermore, we hypothesised that the amount of oligo(dT) beads could influence RNA recovery and, if in excess, promote the capture of unspecific binders. Indeed, while higher amounts of oligo(dT) resulted in an increase in recovered RBPs, it also caused a concomitant increase in proteins in the non-irradiated control (Figure S2B). We observed that 250 μL of oligo(dT) magnetic beads per sample resulted in the optimal signal-to-noise ratio.

To further reduce the incidence of potential unspecific binders, we introduced an extra wash step with a buffer that we refer to herein as ‘harsh’, which included higher concentrations of the ionic detergent LiDS (1% *wt*/*v*) and of LiCl (2M). The inclusion of this wash step was expected to fully dissociate non-covalent interactions that might remain attached to the beads or oligo(dT) probe throughout the RNA capture process. By applying these modifications, we increased the proportion of proteins enriched in UV-irradiated samples over controls to 89% and, as a consequence, the proportion of +UV/−UV non-enriched proteins dropped to 11% (Figure 2C,D). These numbers correlate well with previous RIC experiments performed in mammalian cells [13].

**Figure 3:**
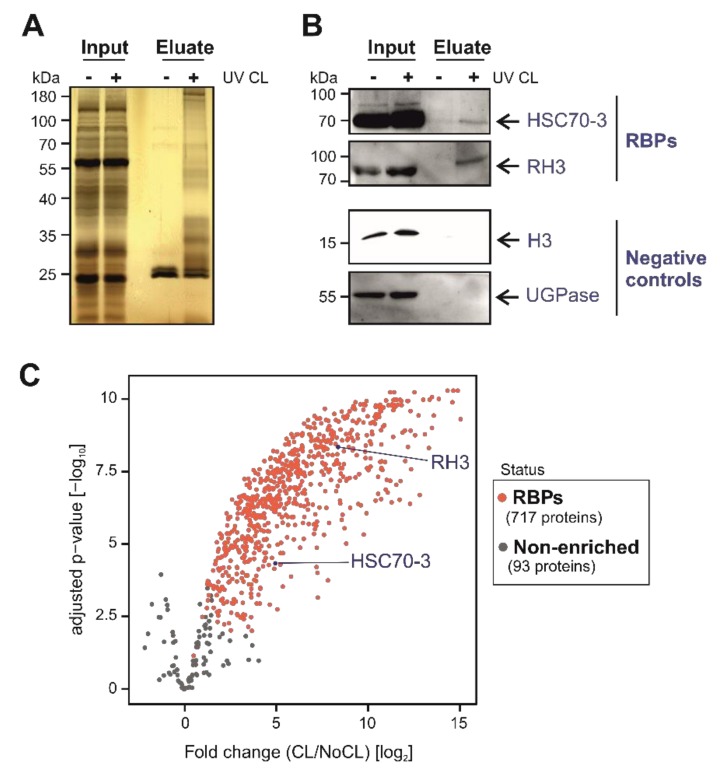
Identification and validation of Arabidopsis leaf RNA-binding proteins (RBPs) using plant RNA interactome capture (ptRIC). (**A**) Silver staining analyses of the inputs (whole cell lysates) and eluates (RBPs) of the ptRIC of Arabidopsis leaves. (**B**) Western blotting analyses of the inputs (whole cell lysates) and eluates (RBPome) of the ptRIC using antibodies against RNA HELICASE 3 (RH3), the chaperone HEAT SHOCK COGNATE PROTEIN 70-3 (HSC70-3), UDP-GLUCOSE PYROPHOSPHORYLASE (UGPase) and HISTONE 3 (H3). “+” and “−“ indicate ‘+ UV crosslinking’ or ‘−UV crosslinking’. (**C**) Volcano plot depicts the log_2_ fold change (log_2_ FC(CL/NoCL)) and the significance (−log_10_ adjusted *p*-value) between no UV crosslinking (NoCL) and UV crosslinking (CL) treatments for each protein (dots) using data from four biological replicates. The colour of the dots indicates if proteins were classified as ‘RBPs’ (red) or +UV/−UV ‘non-enriched proteins’ (grey).

Silver staining analyses of the proteins captured with the optimised ptRIC protocol revealed a specific pattern in UV-irradiated samples that resembled that of previously established RBPomes (Figure 3A) [4,13]. Importantly, the lane of the non-irradiated control eluate was highly depleted of proteins, confirming the stringency of our purification (Figure 3A). Moreover, the banding pattern of the UV-irradiated eluate strongly differed from the input (whole cell lysate), indicating the isolation of a specific subset of proteins, likely to be RBPs (Figure 3A).

### 3.2. ptRIC Captures Bona Fide RBPs

To further validate these results, we used ptRIC followed by Western blotting focusing on specific proteins. We confirmed that RNA HELICASE 3 (RH3) and the chaperone HEAT SHOCK COGNATE PROTEIN 70-3 (HSC70-3), which were classified as RBPs by ptRIC, were enriched in eluates in a UV-dependent manner (Figure 3B). Several chaperones have been reported as RBPs in mammalian systems, fruit fly and yeast [12,32], and have also been identified in plants [32]. Hence, this represents an example of an unconventional RBP conserved across eukaryotes. As negative controls, we used the cytoplasmic marker UDP-GLUCOSE PYROPHOSPHORYLASE (UGPase), which is absent in the RBPome, and HISTONE 3 (H3) as a proxy for DNA contamination. Both proteins were detected in the inputs (whole cell lysates) but were absent in eluates, showing the high stringency of our improved RIC protocol (Figure 3B).

### 3.3. Building a High-Confidence Leaf RBPome for Arabidopsis

To establish the Arabidopsis leaf RBPome, we calculated the log_2_ fold change between UV crosslinked and non-crosslinked samples (log_2_FC (CL/NoCL)) for each protein and estimated the false discovery rate (FDR) of each change using data from four biological replicates. We classified proteins as high-confidence RBPs when the log_2_FC (CL/NoCL) > 1.5 and the adjusted *p*-value (FDR) < 0.01. Following this method, 427 proteins were classified as high-confidence Arabidopsis leaf RBPs. However, a large number of proteins identified had missing values (i.e., zero intensity) in all the NoCL controls (Figure S1B), which resulted in ‘infinite’ ratios that, in principle, could not be handled statistically. The high incidence of ‘zero’ intensity values in the negative control highlighted the high stringency of our experimental approach in plant tissue, when compared to the original protocol (Figure 2, Figure S1B). To discover RBPs with missing values in the negative control, we used a modification of the semi-quantitative method described by Sysoev [2]. This classified as RBPs proteins with intensity values in (at least) three out of four CL samples and lacking signal in all four non-irradiated controls (NoCL). This analysis resulted in 290 additional proteins being classified as RBPs.

Overall, our improved RIC protocol and the combination of quantitative and semi-quantitative data analyses led to the identification of 717 high-confidence RBPs in Arabidopsis leaves (Figure 3C; Table S1). Our protocol outperforms previously plant-adapted RIC protocols [34,35,37] and can now be adapted to other plant tissues such as roots or flowers and, potentially, to microorganisms or animal organs. However, application of ptRIC to leaves of other plant species and other tissues may require additional optimisation. Importantly, ptRIC can now be applied to elucidate how RBPs contribute to remodelling gene expression during physiological processes such as development, or in response to environmental and pathological cues.

### 3.4. Function and Localization of Leaf RBPs

**Figure 4:**
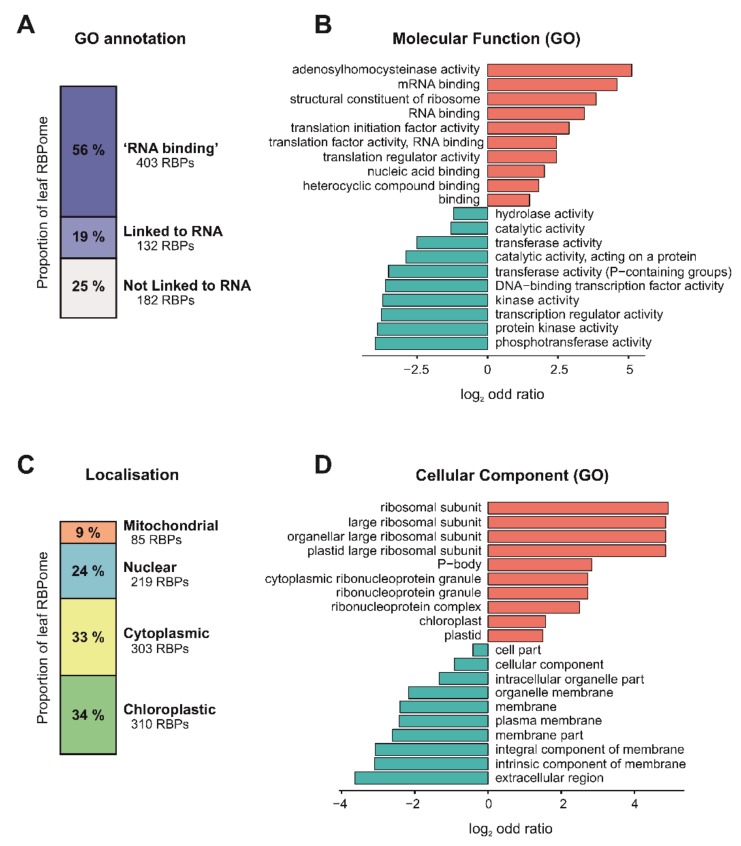
Insights into the Arabidopsis leaf RNA-binding proteome (RBPome) identified by plant RNA interactome capture (ptRIC). (**A**) Proportions of the leaf RBPome with the gene ontology (GO) annotation ‘RNA binding’, GO annotations ‘linked to RNA biology’ or with no GO annotations ‘linked to RNA biology’. GO analysis showing ten of the most significantly enriched (red) or underrepresented (blue) molecular function (**B**) or cellular component (**D**) GO terms for the leaf RBPome. (**C**) Proportions of the leaf RBPome localised to mitochondria, nucleus, cytoplasm and chloroplast based on GO annotations.

We next investigated whether the high-confidence leaf RBPome had known links to RNA biology using gene ontology (GO) annotations. Importantly, approximately 56% of the leaf RBPs were annotated as ‘RNA binding’, and an additional 19% had annotations linked to RNA biology (Figure 4A), indicating that ptRIC is an effective technique to uncover high-quality RBPomes. For example, we identified the well-characterised RBPs AGO1 and AGO2, which are involved in RNAi [47]. Interestingly, the remaining 25% RBPs had no known or predicted role in RNA biology (Figure 4A) and thus represent potentially novel RBPs. The proportion of these novel RBPs was similar to that in the RBPome of etiolated seedlings and protoplasts (42%) [34,37], yeast (42%) or humans (31%) [21]. ‘RNA binding’, ‘mRNA binding’ or ‘nucleic acid binding’ were amongst the most enriched GO terms for the leaf RBPome, further confirming the validity of our approach (Figure 4B). Other significantly enriched GO terms included other RNA-related functions such as ‘structural constituent of ribosome’, ‘translation initiation factor activity’ and ‘translation regulator activity’. The most enriched GO term was ‘adenosylhomocysteinase activity’. Interestingly, the Arabidopsis S-Adenosyl-L-Homocysteine Hydrolase HOG1 is involved in transcriptional gene silencing and DNA methylation [48], although its potential roles in RNA metabolism are currently unknown. We found amongst the statistically underrepresented terms RNA-unrelated functions such as ‘phosphotransferase activity’, ‘protein kinase’ and ‘DNA-binding transcription factor’. The depletion of DNA-binding proteins in the leaf RBPome indicated that our eluates were largely depleted of DNA. These results are similar to those previously observed in mammalian RIC experiments [13].

The leaf RBPs were annotated to be localised not only in the nucleus (219 RBPs) or cytoplasm (303 RBPs), but also in the chloroplast (310 RBPs) and mitochondria (86 RBPs), indicating that ptRIC allows deep analyses of the Arabidopsis RBPome, including discovery of RBPs from different subcellular compartments and organelles (Figure 4C). When compared to the reference Arabidopsis proteome, ptRIC identified only a small subset of proteins for each of the subcellular compartments (3–9%) (Figure S3). This was not unexpected since, for example, the human RBPome has been estimated to comprise about 7.5% of the total cellular proteome [11].

Interestingly, we identified larger numbers of RBPs localising to the chloroplasts than anticipated (Figure 4C). Accordingly, ‘chloroplast’ and ‘plastid’ were statistically enriched GO cellular component terms (Figure 4D). The intensity of many of these chloroplastic proteins in the RIC eluates was similar to that of RBPs harbouring classic RBDs (Figure S4A,C), suggesting that these organellar proteins crosslink efficiently to RNA. In plants, chloroplastic and mitochondrial RNAs can be polyadenylated, and addition of poly(A) tails in these plant organelles is a tag for RNA degradation [49]. It is also known that certain conditions, such as light deprivation, promote polyadenylation of chloroplastic RNAs [50]. Hence, chloroplastic RBPs captured by ptRIC could be bound to polyadenylated RNAs that are undergoing degradation in these organelles. Alternatively, these RBPs may also possess extra-organellar functions and be associated with cytoplasmic or nuclear RNAs with stable poly(A) tails.

### 3.5. RNA-Binding Domains in Leaf RBPs

**Figure 5:**
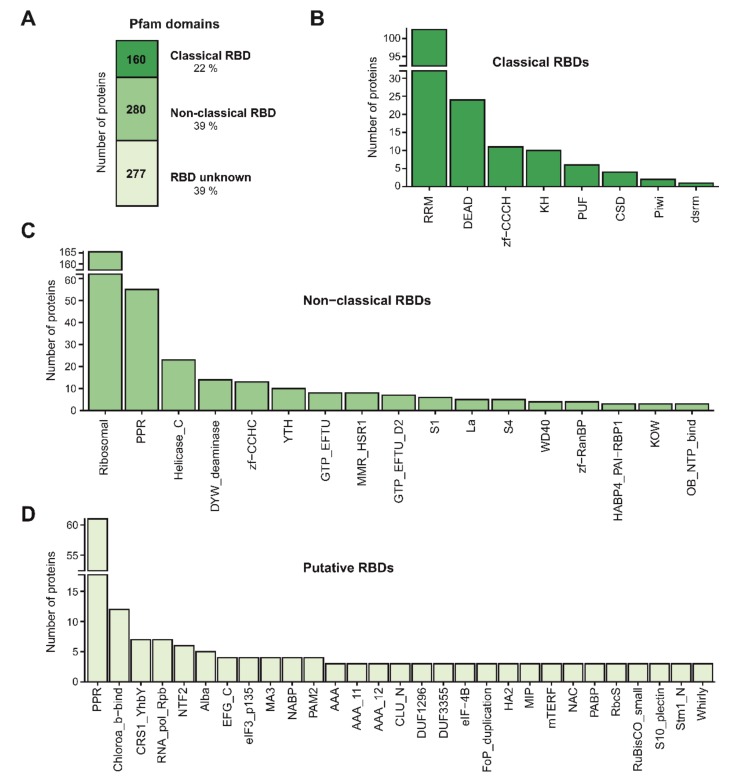
Domain architecture of the Arabidopsis leaf RNA-binding proteome (RBPome). (**A**) Number of proteins harbouring classical, non-classical or no known RNA-binding domains (RBDs) in the leaf RBPome based on Pfam annotation. (**B**) Number of proteins annotated as possessing classical RBDs, (**C**) non-classical RBDs or (**D**) putative RBDs. For non-classical and putative RBD, only RBDs with at least three counts are shown.

We expected a strong enrichment in previously characterised RNA-binding domains (RBDs) if our RBPome was enriched in bona fide RBPs. In agreement, based on Pfam annotations about 60% of the leaf RBPs harboured known RBDs (classical or non-classical RBDs), whereas the remaining 40% harboured no recognisable RBDs (Figure 5A). These proportions are similar to RBPomes generated in other species including humans and plants [13,34,35]. Classical RBDs are domains well-characterised at the biochemical and structural level [51], whereas we refer to protein domains that have been described to bind to RNA in literature at least once as non-classical RBDs, although their interactions with RNA are in many instances not well-established biochemically and/or structurally. As expected, 160 RBPs harboured classical RBDs, the most prominent being the RNA recognition motif (RRM), DEAD-box helicase, zinc finger (zf)-CCCH and K-homology domains (KH) (Figure 5B). In agreement, these are the most prevalent RBDs in both plants and mammals [13,34]. For example, the leaf RBPome contains 103 RRM-containing proteins out of the 253 found in the Arabidopsis reference proteome, 24 out of 96 DEAD-containing proteins, 11 out of 47 zf-CCCH-containing proteins and 10 out of 32 predicted KH-containing proteins. Moreover, ptRIC identified all four cold shock domain (CSD)-containing proteins.

We identified 280 RBPs of which the RNA-binding activity could be explained by non-classical RBDs. These included ribosomal, pentatricopeptide repeat (PPR), helicase C-terminal and zf-CCHC as the most prominent domains (Figure 5C). The fact that a large proportion of non-classical RBDs are ribosomal has been observed in the RBPomes of other organisms [13,27,34]. This is not surprising, since it is known that each ribosomal protein assembles with its target RNA sequence via an intimate co-folding process into the ribosome nano-machinery, thus possessing protein-specific modes to interact with rRNA. Ribosomes sit on the translating mRNA and the ribosomal proteins within the mRNA channel are overrepresented in RBPomes, reflecting the fact that those proteins are the most likely to crosslink to polyadenylated RNA during the translation process [52,53]. However, other ribosomal proteins are detected in the RBPome in sub-stoichiometric quantities [53]. These can proceed from extra-ribosomal, regulatory interactions with mRNA [54] or can be structural ribosomal proteins isolated through crosslinking to non-polyadenylated rRNA [53]. We also identified 10 of the 13 YT521-B homology (YTH)-containing proteins in Arabidopsis [55], which are known to ‘read’ methyl 6 adenosine modifications within RNA [56]. YTH-containing proteins were previously classified as plant RBPs in the RBPome of Arabidopsis seedlings [34], and we confirmed their RNA-binding activity here.

The PPR-containing protein family has expanded in plants as compared to metazoans (e.g., 450 PPR-containing proteins in Arabidopsis vs. 8 in humans) [57,58] and many plant PPR-containing RBPs are exclusive to plants and seem to have evolved from expansion of a small subset of eukaryotic conserved PPR-containing proteins [59]. Some PPR subclasses are known to engage in interactions with RNA [60] and are classified as ‘non-classical RBDs’, whereas for other subclasses, there are no established links to RNA biology, so they are classified as ‘putative RBDs’. ptRIC identified 55 RBPs containing ‘non-classical RBD’ PPR subclasses, but also 61 RBPs containing PPR subclasses that lack links to RNA biology. This contrasted with the low numbers identified in other plant RBPomes, ranging from 8 to 24 PPR-containing proteins [34,35,37]. The fact that most PPR-containing proteins localise to mitochondria and chloroplasts [60], and that we identified larger numbers of PPR-containing proteins than previous studies, indicates that ptRIC allows deep characterisation of organellar RBPs. Other domains such as the C-terminal DYW or E domains are often present with the PPR domains in proteins [59]. We identified 14 RBPs containing the DYW domain, which contributes to specific recognition of the RNA editing sites [61] and has been proposed to be the catalytic domain for RNA editing [62,63].

A large proportion of the identified RBPs did not possess any recognisable RBD (277 RBPs; ~40% of the leaf RBPome; Figure 5A,D). We evaluated the likelihood of these proteins being genuine RBPs by performing a GO enrichment analysis. Strikingly, the most enriched GO terms were related to ‘RNA biology’, including ‘RNA binding’, and ‘translation initiation factor activity’ (Figure S5). This indicated that although 40% of the leaf RBPs did not possess known RBDs, they were likely to be RBPs. These results raise questions about how these unconventional RBPs interact with RNA.

For example, the leaf RBPome contained three RBPs harbouring the DUF1296/GBF-interacting protein 1 (GIP1) domain. Moreover, proteins containing the DUF1296/GIP1 domain were also identified in the RBPome of etiolated Arabidopsis seedlings [34]. Hence, these results support the role of DUF1296/GIP1 as a putative RBD in plants. ptRIC also assigned RNA-binding activity to all the members of the Alba- and the Whirly (WHY)-domain-containing protein families. These proteins were also found to interact with RNA in the RBPome of etiolated seedlings [34], which strongly suggest that these domains interact with RNA in plants. We identified two out of three multiprotein bridging factor 1 (MBF1)-containing proteins. MBF1 proteins are transcriptional coactivators that are found in a wide range of archaea and eukaryote species, from fungi to metazoans and plants [64]. However, contrarily to many other eukaryotes, plants possess multiple genes encoding MBF1 proteins instead of one [64]. Although the MBF1 protein from *Drosophila* and yeast can bind RNA [65,66], to our knowledge, this domain has not been previously linked with RNA binding in plants. Our data suggests that the MBF1 domain is a novel RBD in plants. We also identified three proteins that contained the major intrinsic protein (MIP) domain. The MIP domain is present in proteins belonging to a large family of transmembrane channels (including aquaporins) that transport water, small molecules, ions and gases [67,68,69]. Although aquaporins have been postulated to bind RNA in Arabidopsis by previous RIC analyses [34], their RNA-binding activity remains to be experimentally validated by orthogonal approaches. Strikingly, we identified 12 RBPs possessing the Chlorophyll A-B binding domain and additional proteins containing 15 different photosynthesis-related domains, such as PsaA_PsaB, PsaD, PsaL and PsbH, in our leaf RBPome (Figure 5D). This suggests that proteins of the photosynthetic apparatus moonlight as RNA binders, as further discussed below.

Hence, domains that are enriched in RBPs but lack previous links to RNA biology could represent novel RBDs. However, further experiments have to be undertaken to determine if these domains are endowed with RNA-binding activity. Alternatively, RBPs that do not possess known RBDs could also bind RNA through disordered regions in analogy to other eukaryotic systems [11]. Recent proteome-wide analyses of RBDs have discovered that hundreds of proteins in the human proteome interact with RNA via intrinsically disordered regions (IDRs), which are protein regions lacking 3D structure in their native state [11,12]. RNA-binding IDRs are enriched in amino acids typically found at the protein–RNA interfaces in globular domains, including tyrosine, arginine and lysine. IDRs can be found in RBPs as the sole RNA-binding motif or can cooperate with globular RBDs [12,13]. The contribution of IDRs to RNA binding in plants deserves further characterisation.

### 3.6. RBPs Uniquely Identified by ptRIC

**Figure 6:**
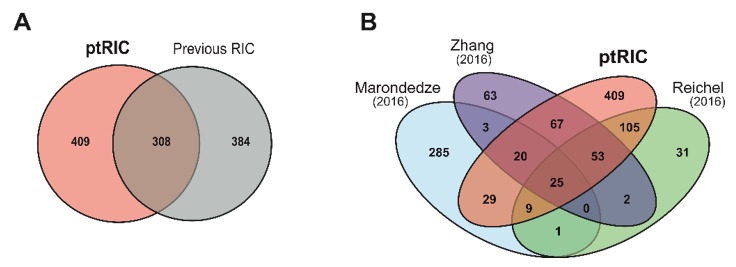
Overlap between the present plant RNA interactome capture (ptRIC) results and previous RNA interactome capture (RIC) studies. Venn diagrams depict the overlap between the ptRIC leaf RNA-binding proteome (RBPome) and the three previously published Arabidopsis RBPomes together (**A**) or individually (**B**). Each of the previous RIC studies used different plant tissues: Marondedze and colleagues used cell cultures and leaves, Zhang and colleagues used mesophyll protoplasts and Reichel and colleagues used etiolated seedlings.

Next, we compared our leaf RBPome with those previously published for Arabidopsis [34,35,37]. We used the data shown in Hentze et al. [32], as it compiled all the published plant RBPomes using a unified analytical criteria. Collectively, the previous plant RBPome studies identified 719 RBPs using different Arabidopsis tissues. By applying ptRIC to leaf tissue, we discovered 717 RBPs, 409 of which (57%) were not identified in the previous RIC studies (Figure 6A,B). These RBPs may represent leaf- or developmental-stage-specific RBPs. Alternatively, these RBPs may have been present in our dataset simply because our improved protocol allowed deeper RBPome analysis due to its higher signal-to-noise dynamic range. Surprisingly, there were only 25 RBPs shared between the four RBPomes. This number was lower than expected, especially when considering that hundreds of RBPs are shared between yeast and humans [21]. This divergence could be due to the different nature of the starting materials (i.e., cell cultures, mesophyll protoplasts, etiolated seedlings and leaves of mature plants) and, more plausibly, to differences in the protocol, including the proteomics analysis and the stringency of downstream analytical workflow. Importantly, the RBPomes of mesophyll protoplasts, etiolated seedlings and leaves (this study) overlap substantially, suggesting that the RBPome of cultured cells is an outlier (Figure 6B, Figure S6). One example of an unconventional RBP shared by all plant RBPomes is CATALASE-3. The enzyme catalase has only been described to associate with RNA in cows [70], but the consensus between datasets in identifying this enzyme as being associated with RNA provides strong evidence for a moonlighting function as an RBP in plants.

We also tested whether the large number of ptRIC-specific RBPs was due to a more efficient capture of RBPs from subcellular compartments. To test this, we constructed a superset of the Arabidopsis RBPs identified in previous studies [32] and evaluated this superset against the leaf RBPome. No statistical enrichment or depletion of GO cellular component was found. These results indicated that ptRIC did not enhance RBP recovery from a given organelle, but increased the overall depth of the RBPome analysis. Nevertheless, we noticed a substantial proportion of mitochondrial and chloroplastic RBPs, implying a high RBP coverage for these subcellular organelles (Figure 4C, Figure S3).

To obtain deeper insights into the 409 plant RBPs exclusively identified here, we analysed their domain distribution across known and newly discovered RBPs (Figure S7D–G). We discovered that RBPs exclusively identified by ptRIC displayed similar domain composition to those identified by multiple plant RBPomes (Figure 5) [34,35,37]. Moreover, gene set enrichment analysis using GO (Figure S7A,B) revealed similar GO annotations to be enriched in both RBPs exclusively identified here and those previously reported by other RIC experiments (Figure 4B,D) [34,35,37]. These GO terms included ‘mRNA binding’, ‘ribonucleoside binding’ or ‘RNA binding’ (Figure S7A). Other RNA-related functions were also statistically enriched, such as ‘structural constituent of ribosome’ (Figure S7A). Similarly, the statistically underrepresented terms were also ‘phosphotransferase activity’, ‘protein kinase’ activity and ‘hydrolase activity’ (Figure S7A).

Importantly, 42% of the leaf ptRIC-specific RBPs are annotated as ‘RNA binding’ and an additional 25% had previously been linked in some extent to RNA biology (Figure S7C). Hence, 33% of the RBPs discovered uniquely by ptRIC had not been previously associated with RNA biology (Figure S7C) and represent potential novel RBPs. This contrasts with the set of 285 proteins exclusively identified by Marondedze and colleagues [35], of which only 18% were annotated as ‘RNA binding’ or linked to RNA biology, and which were enriched in GO terms largely unrelated to RNA biology. Taken together, these results strongly support that the proteins solely identified by ptRIC are enriched in *bona fide* RBPs.

### 3.7. Unconventional RNA-Binding Proteins in Plant Leaves

Moonlighting functions have been described for a number of metabolic enzymes across eukaryotes, including plants [21,23,35,71]. For example, the leaf RBPome included two chloroplastic glyceraldehyde-3-phosphate dehydrogenases (GAPDH), two fructose-bisphosphate aldolases and TRANSKETOLASE, enzymes that have been previously reported to bind RNA in other organisms by RIC [13,32,34,35]. GAPDH is a good example of a well-studied metabolic enzyme that moonlights as an RBP. GAPDH catalyses the conversion of glyceraldehyde-3-phosphate to D-glycerate-1,3-bisphosphate and generates NADH. Moreover, GAPDH binds to a variety of RNAs from mRNAs to rRNAs and viral RNAs [71]. For example, GAPDH regulates T-cell effector function by moonlighting between glycolysis and binding to the 3′ UTR of mRNAs encoding cytokines [72]. Moreover, ptRIC also identified a phosphoglycerate kinase and two thioredoxins, which have been validated to bind RNA in humans and yeast [12,21]. Thus, these metabolic enzymes have conserved RNA-binding activities from yeast to humans and plants.

Additional moonlighting enzymes were identified in the leaf RBPome, such as COBALAMIN-INDEPENDENT METHIONINE SYNTHASE, (S)-2-HYDROXY-ACID OXIDASE, GLYCINE DEHYDROGENASE and GLUTAMATE-GLYOXYLATE AMINOTRANSFERASE, which, to our knowledge, have not previously been linked to RNA biology (Table S1). Moreover, we identified several peptidases that associated with RNA such as METHIONINE AMINOPEPTIDASE 1B, which excises the N-terminal methionine from nascent peptides [73]. It is of note that methionine aminopeptidases have previously been identified in the RBPomes of multiple species, including human, mouse and yeast [32]. Some proteases are known to bind RNA, as illustrated by the human mitochondrial Lon protease [74]. However, the extent to which RNA-binding activity is present in proteases and its functional implications remains largely unknown. With the use of RIC, it has become evident that the moonlighting function of some metabolic enzymes as RBPs is a widespread phenomenon, and it has been shown that the RNA-binding activity of several of these metabolic enzymes is crucial for cell biology [71]. However, additional functional studies are required to understand the crosstalk between metabolism and gene regulation. It will be particularly interesting to study the RNAs regulated by metabolic enzymes and whether the interaction with RNA alters their enzymatic activity. It will also be crucial to determine whether the RNA-binding activity of metabolic enzymes is modulated in a cellular-state-dependent manner, as has been described for the human aconitase 1 or iron regulatory protein 1 [75].

### 3.8. Proteins of the Photosynthetic Apparatus Moonlight as RNA Binders

We observed that about 8% of the leaf RBPs (56 proteins) had annotations related to photosynthesis or photosystems (Table S2), and of these, 23 were exclusively identified here. In agreement, many domains related to photosynthesis were also enriched in the leaf RBPome, including Chloroa_b-bind, which is present in proteins that belong to the light-harvesting complex (LHC), and RuBisCO_small (Figure 5D). Leaf RBPs include proteins from the four major protein photosynthetic supercomplexes: photosystem II (PSII) and I (PSI), the cytochrome b6f and F-ATPase [76]. For example, we identified 18 proteins from PSII, 13 from PSI, 3 from the cytochrome b6f complex and 6 from the F-ATPase complex to be associating with RNA (Table S2). We also found that additional photosynthesis-related proteins such as the RUBISCO large subunit and small subunits 1A, 2A and 2B were associated with RNA in vivo.

A priori this may seem surprising, since photosynthesis has not been extensively linked to RNA metabolism. However, there are multiple lines of evidence that support the hypothesis that components of the photosynthetic apparatus can associate with RNA. Firstly, the intensity of most of these photosynthetic proteins in ptRIC was relatively high, suggesting that these were bona fide RBPs (Figure S4B). Secondly, 33 leaf RBPs involved in photosynthesis have also been independently shown to associate with RNA in other Arabidopsis tissues by RIC analyses [32,33], with the exception of seedlings grown in the dark [34]. Thirdly, five leaf RBPs with GO annotations related to photosynthesis were also annotated as ‘RNA binding’ (OEE1, OEE2, HPR1, WTF1 homolog and the uncharacterised PPR-containing protein AT3G46610). Lastly, some photosynthesis components, such as the large subunit of rubisco (LSU) and cytochrome f, are known to bind RNA. The LSU of *Chlamydomonas reinhardtii* contains an RRM domain at the N-terminus that becomes exposed under oxidising conditions and triggers its capacity to bind RNA [77,78,79]. The structure of this N-terminal domain is highly conserved throughout evolution, despite low sequence similarity between species, and its RNA-binding activity has been experimentally validated in different photosynthetic organisms [78]. Moreover, cytochrome f of *Chlamydomonas reinhardtii* regulates its own RNA translation by binding the 5′UTR via the C-termini of the protein [80].

Given the evidence that photosynthetic proteins may associate with RNA, it is tempting to speculate that components of the photosynthetic apparatus may act as RNA regulators similarly to moonlighting metabolic enzymes [71]. Alternatively, these proteins could be allosterically regulated by RNA binding in a new case of ‘riboregulation’. Recent studies have provided evidence that RNAs can play a role in regulating proteins, such as the small non-coding vault1-1 RNA on the component of the autophagy pathway p62 [81], and also have other important roles such as serving as a scaffold to recruit, organise or sequester proteins within complexes [32]. However, orthogonal approaches are required to confirm the RNA-binding activity of these components of the photosynthetic apparatus, and understand its biological role. For example, further studies to identify the RNAs bound by these proteins and the biological consequences of these protein–RNA interactions will shed light on how photosynthesis regulates or is regulated by RNA.

## 4. Conclusions

We improved RIC to perform efficiently in plant leaves. This modified protocol, referred to here as ‘plant RNA interactome capture’ (ptRIC), has generated a comprehensive Arabidopsis leaf RBPome, which includes 717 proteins. While 75% of the RBPs identified by ptRIC had known or predicted links to RNA biology, 25% were previously unknown to interact with RNA, offering new avenues of research to the plant community. Notably, ptRIC captured several metabolic enzymes interacting with RNA, and showed that the photosynthetic apparatus engages with RNA through several of its components. Moreover, 39% of the identified RBPs lacked known RBDs, suggesting the existence of novel modes of RNA binding that remain to be discovered. The existence of still uncharacterised RBDs spans from yeast to human and plants, reinforcing the importance of this phenomenon [21]. Because ptRIC allows deep analysis of the RBPome of plant leaves, we foresee that it will be an excellent approach for the study of the dynamics of plant RNA-binding proteins in response to different environmental, physiological and pathological conditions. These studies will shed light on how plants adapt their RNA metabolism and gene expression in response to a changing environment.

## Supplementary Materials

The following are available online at https://www.mdpi.com/2218-273X/10/4/661/s1, Figure S1: Raw intensities are used for protein analyses, Figure S2: Optimisation of multiple parameters of ptRIC, Figure S3: Localisation of leaf RBPs, Figure S4: Chloroplastic and photosynthetic proteins are true RBPs, Figure S5: Annotation of RBPs lacking known RNA-binding domains (RBDs), Figure S6: Overlap between the ptRIC leaf RBPome and previous Arabidopsis RBPomes, Figure S7: Insights into the ptRIC-specific leaf RBPs, Table S1: Leaf RBPs identified by ptRIC, Table S2: Proteins from photosynthetic apparatus identified in leaf RBPome, One Supplementary File including a Step-by-step ptRIC protocol, One Supplementary File including Supplementary Materials and Methods.
