# Supplementary material for: Discovering the RNA-Binding Proteome of Plant Leaves with an Improved RNA Interactome Capture Method
Source: Biomolecules. 2020 Apr 24;10(4):661. doi: 10.3390/biom10040661 (PMC7226388; doi:10.3390/biom10040661)
Supplement: Supplementary file 1 [file biomolecules-10-00661-s001.zip › biomolecules-776768-supplementary-final version/Step_by_step_protocol.pdf]

Plant RNA interactome capture (ptRIC) protocol

Marcel Bach-Pages

1. Introduction

RNA interactome capture in plants can be challenging due to the nature of plant tissue as compared to human cell lines. For example, plants contain pigments such as chlorophylls that can reduce the efficiency of UV crosslinking. Additionally, leaves can introduce further challenges, since its thickness reduces crosslinking efficiency. Furthermore, the composition of leaves is more complex than human cell lines due to the presence of the cell wall and additional secondary metabolites [1]. The following protocol has been optimised for mature, fully expanded leaves of *Arabidopsis thaliana.* Further optimisation may be required for other tissues or other plant species.

2. Plant material

Grow *Arabidopsis thaliana* plants for 5-6 weeks (Col-0 ecotype) in soil at neutral day conditions (12 h light, 12 h dark) at 20 °C and light intensity of approximately 100 µmol/m^2/s.

3. Buffer preparation

3.1. Prepare, filter and store the solutions at 4°C for up to 3 months. IGEPAL, Polyvinylpyrrolidone (PVP40), ß-mercaptoethanol (B-ME), DTT (Dithiothreitol), and the Protease and RNase inhibitors are immediately added before use. Use ultrapure water to prepare the buffers.

- Lysis buffer: 20 mM Tris-HCl (pH 7.5), 500 mM LiCl, 1 mM EDTA, 0.5% LiDS (wt/v), 0.02% IGEPAL (v/v), 2.5% PVP40 (wt/v), 1% B-ME (v/v), 5mM DTT, protease inhibitor and RNase inhibitor.
- Harsh buffer: 20 mM Tris-HCl (pH 7.5), 2 M LiCl, 1 mM EDTA, 1% LiDS (wt/v), 0.02% IGEPAL (v/v), 5mM DTT.
- Buffer I: 20 mM Tris-HCl (pH 7.5), 500 mM LiCl, 1 mM EDTA, 0.1% LiDS (wt/v), 0.02% IGEPAL (v/v), 5mM DTT.
- Buffer II: 20 mM Tris-HCl (pH 7.5), 500 mM LiCl, 1 mM EDTA, 0.02% IGEPAL (v/v), 5mM DTT.
- Buffer III + IGEPAL: 20 mM Tris-HCl (pH 7.5), 200 mM LiCl, 1 mM EDTA, 0.02% IGEPAL (v/v), 5mM DTT.
- Buffer III NO IGEPAL: 20 mM Tris-HCl (pH 7.5), 200 mM LiCl, 1 mM EDTA, 5mM DTT.

- Elution buffer: 20 mM Tris-HCl (pH 7.5), 1 mM EDTA.

  Note: The catalogue numbers for each of the reagents are the following: Tris (Sigma-Aldrich, cat. no. T1503), LiCL (Sigma-Aldrich, cat. no. 62476), EDTA (Sigma-Aldrich, cat. no. E5134), LiDS (Sigma-Aldrich, cat. no. L4632), IGEPAL (Sigma-Aldrich, cat. no. I3021), PVP40 (Sigma-Aldrich, cat. no. PVP40), B-ME (Sigma-Aldrich, cat. no. M6250), DTT (Thermo Scientific, cat. no. R0862), Protease Inh. (Roche, cat. no. 11873580001), RNase inh. (Promega N2611).

  Note: plant tissues contain high levels of RNases [1], hence adding RNase inhibitors is recommended. Plant cells also contain high levels of secondary metabolites that require the use of components such as PVP40 to complex polysaccharides and polyphenols.

4. UV crosslinking

4.1. Excise and place leaves of mature Arabidopsis plants on a plastic sheet on top of ice pads and UV crosslink three times (twice on the adaxial side of the leaves and once on the abaxial side) with 150 mJ/cm^2 of UV light at 254 nm wavelength using a Stratalinker 2400 (Stratagene cat. no. 400076). Allow 30 seconds pause in between irradiations.

  Note: UV-crosslinking creates short-lived highly reactive states within the RNA/DNA nucleotides that react with molecules that are in intimate contact with them. Thus, UV irradiation promotes formation of covalent bonds between RNAs and the proteins (RBPs) intimately associated with them [2]. Crosslinking both sides of the leaf ensures uniformity in crosslinking throughout the leaf.

4.2. For the non-crosslinked (NoCL) negative control, place the leaves on ice for approximately the same time as the crosslinked (CL) samples (~3 minutes).

4.3. Freeze the leaves immediately in liquid nitrogen to preserve the molecular interactions and sample integrity.

5. Cell lysis

5.1. Grind leaf tissue to a fine powder in liquid nitrogen using a mortar and pestle.

5.2. Mix 1.2 g of tissue with 12 mL of lysis buffer in a 50 ml tube. Keep the lysates on ice to minimise RNA degradation.

5.3. Further homogenise the lysates for 1 minute using a Potter-Elvehjem homogenizer, while keeping the lysates on ice to avoid sample overheating.

5.4. Clear the lysates by centrifugation (4000 rpm, 10 min, 4 °C) and filter the supernatant through Miracloth (Merk, cat. no. 475855).

5.5. Pass the lysates through a narrow needle (27G) five times to shear the gDNA. Clear the lysates again by centrifugation (4000 rpm, 10 min, 4 °C) and filtration of the supernatant through Miracloth.

6. Oligo(dT) capture

6.1. Take aliquots of 200-500 µL of the inputs (whole cell lysates / total proteomes) and store at – 80 °C.

6.2. Activate oligo(dT)$_{25}$ beads (250 µL/sample; NEB, cat. no. S1419S) by washing three times with lysis buffer, using a magnet to trap the magnetic beads followed by supernatant removal.

6.3. Add 250 µL of beads to each of the lysates and incubate at 4 °C for 1 h in a rotator (10 rpm) to allow hybridisation of the oligo(dT) to the poly(A) tail of the RNAs.

Note: longer (even overnight) incubation of the beads with the lysates is also possible.

6.4. Capture the beads on a magnet for ~ 20 - 30 minutes at 4 °C, making sure the supernatant is cleared. Collect the supernatant and store on ice for a second round of capture.

6.5. Wash the beads with 1.5 mL of different buffers with an incubation time of 5 min on ice (except for the wash with harsh buffer, which is performed at room temperature), capture the beads with the magnet and discard supernatant. Invert the tube every 30-60 seconds during incubation time.

- Lysis buffer (three washes).

  Note: the stringency of the lysis buffer allows removal of unspecific binders sticking to the oligo(dT) beads or bound to the RNAs non-covalently or via protein-protein interactions.

- Harsh buffer (one wash at room temperature).

  Note: This washing step is performed at room temperature to increase the removal of unspecific binders and avoid precipitation of the LiDS. The harsh buffer contains higher concentrations of the ionic detergent LiDS (1%, wt/v) and LiCL (2M) to further increase the stringency of the capture.

- Buffer I (two washes).
- Buffer II (two washes).

- Buffer III + IGEPAL (one wash).
- Buffer III NO IGEPAL (one wash).

  Note: IGEPAL can interfere with the downstream mass spectrometry analyses, so the last washing step is performed with a buffer that does not contain this detergent.

6.6. Resuspend the beads with 300 µL of elution buffer and incubate for 3 minutes at 55 °C to elute the RNA-protein complexes.

6.7. Capture the beads using a magnet and transfer the supernatant containing the RNA-protein complexes to a new tube.

6.8. For the second round of capture, wash the beads three times with 1.5 mL of lysis buffer and add to the supernatants recovered from the first capture that were kept at 4 °C. Then proceed with the second round of capture from step 6.3.

Note: only reuse beads for the same condition (i.e. reuse the beads of treatment 1 for the second round of capture of samples of treatment 1).

7. RNA quantification and normalization

7.1. For each of the samples, pool the two eluates from different rounds of capture and quantify using a NanoDrop spectrophotometer (Thermo Fisher Scientific, cat. no. ND1000). Typically, using our settings we obtain RNA concentrations of about 400 - 1000 ng/µL.

7.2. Adjust the volume of the samples so that the same amount of RNA is present in each sample using elution buffer.

Note: An aliquot of 50 µL of the pooled normalised eluates can be separated for RT-qPCR or RNAseq analyses.

8. RNase digestion and protein analyses

8.1. After isolation of the RNA-protein complexes, analyses can be performed on the proteins or the RNAs. For protein analyses of the RNA-protein complexes, treat each of the eluates with 4 µL of RNase A (Sigma-Aldrich, cat. no. R4642) and T1 (Sigma-Aldrich, cat. no. R1003) mix (RNase A and RNase T1 mixed at equal proportions and diluted 1/100) for 1 h at 37 °C followed by incubation for 15 min at 50 °C.

8.2. Concentrate the proteins using an Amicon centrifugal filter of 3 KDa cut-off (Merck, cat. no. UFC500324) by centrifugation (15000 rpm, 4 °C, 1-2h). Measure the volume recovered from each filter (typically between 20 – 30 µL)

and normalise it using elution buffer to proceed with the same volume in each sample

8.3. Samples can be then analysed as following:

- **Western blotting** with specific antibodies.
- **Silver staining** for total protein analyses.
- Quantitative **mass spectrometry** to identify and quantify the isolated proteins. Out of the 600 μL of the eluates, we typically use 50 μL for western blotting and silver staining analyses, and remaining material (550 μL) for mass spectrometry (stored at – 80 °C).

References

1. Köster, T.; Reichel, M.; Staiger, D. CLIP and RNA interactome studies to unravel genome-wide RNA-protein interactions in vivo in *Arabidopsis thaliana*. *Methods* **2019**, 1–9.
2. Pashev, I.G.; Dimitrov, S.I.; Angelov, D. Crosslinking proteins to nucleic acids by ultraviolet laser irradiation. *Trends Biochem. Sci.* **1991**, *16*, 323–326.
