# Supplementary material for: Discovering the RNA-Binding Proteome of Plant Leaves with an Improved RNA Interactome Capture Method
Source: Biomolecules. 2020 Apr 24;10(4):661. doi: 10.3390/biom10040661 (PMC7226388; doi:10.3390/biom10040661)
Supplement: Supplementary file 1 [file biomolecules-10-00661-s001.zip › biomolecules-776768-supplementary-final version/Supplementary_figures.pdf]

Supplementary figures

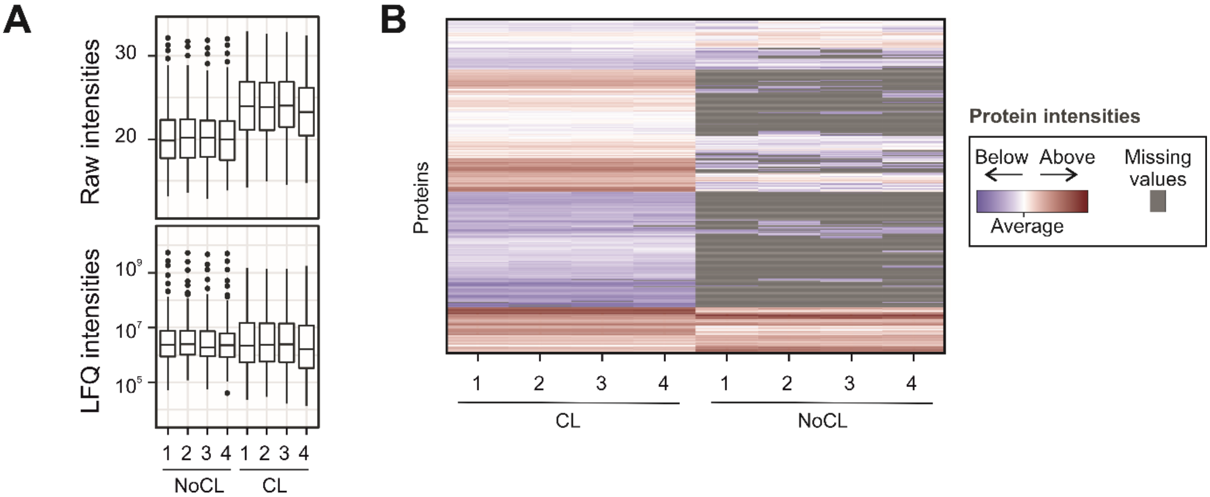

Figure S1. Raw intensities are used for protein analyses. (**A**) Box plots representing the raw intensities or label-free quantification (LFQ) intensities of the proteins identified by plant RNA interactome capture (ptRIC) from each of the no UV crosslinked (NoCL) and UV crosslinked (CL) replicates. (**B**) Heat map representing the profile of intensities of the proteins identified by ptRIC. The raw intensities were re-scaled and intensities above the average are coloured in red, whereas intensities below the average are coloured in blue. Missing values are coloured in grey. Proteins were clustered based on intensity profiles similarity.

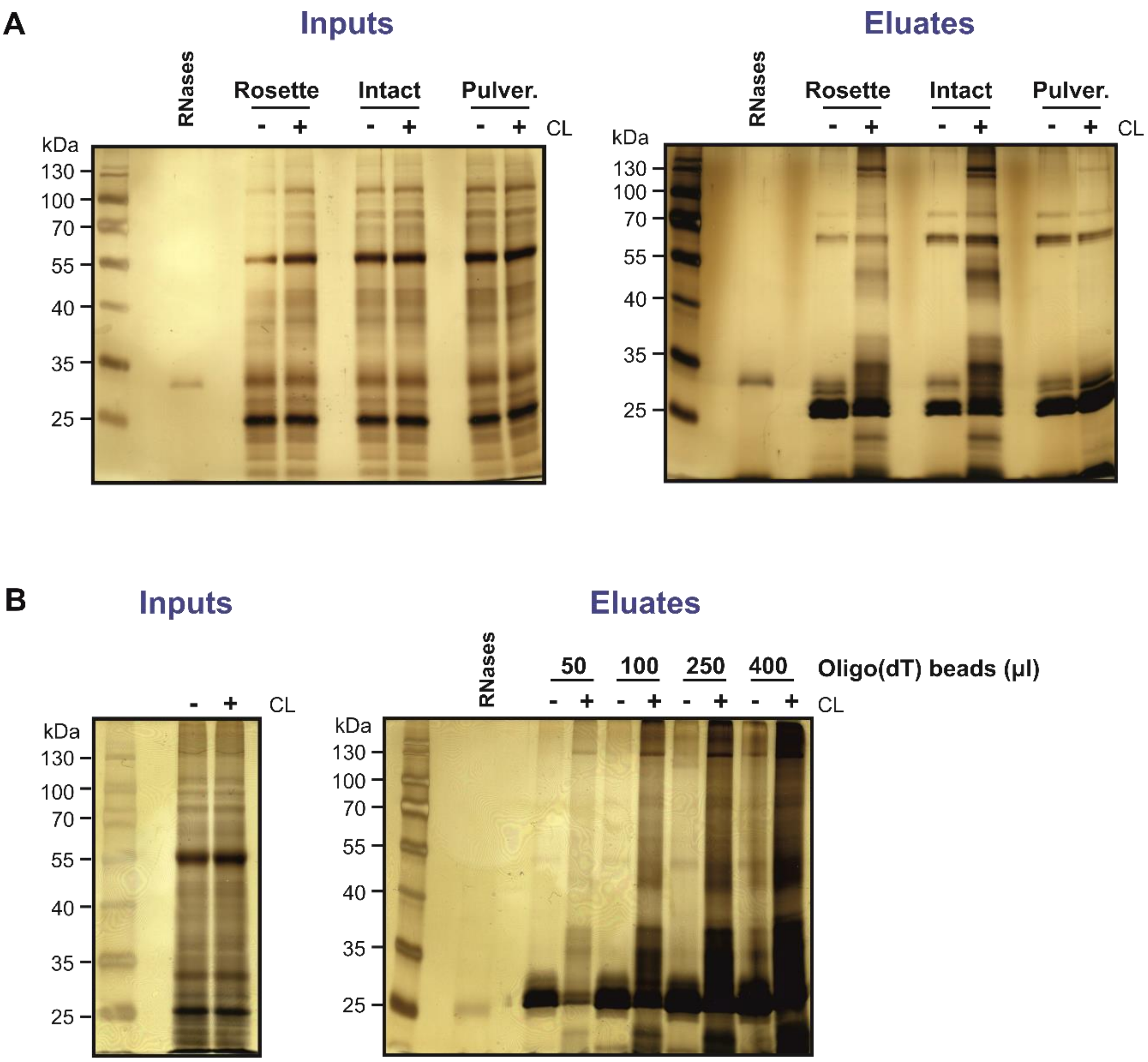

Figure S2. Optimisation of multiple parameters of plant RNA interactome capture (ptRIC). (**A**) Silver staining analyses of the inputs (whole cell lysates) and eluates (RNA-binding proteins) generated during optimisation of ptRIC using full rosettes, intact leaves or pulverised leaves ('pulver') as starting materials. (**B**) Silver staining analyses of the inputs (whole cell lysates) and eluates (RNA-binding proteins) using increasing amounts of oligo(dT) beads, ranging from 50 to 400 µL of beads per sample. '+' and '-' indicate '+ UV crosslinking' or '- UV crosslinking'. The RNases used to digest the RNA in the eluates are loaded as a control.

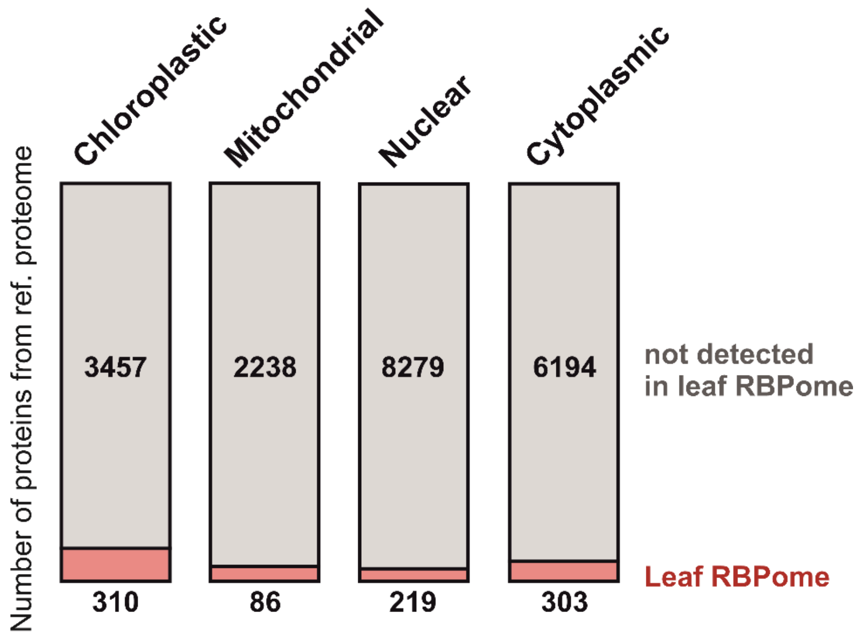

Figure S3. Localisation of leaf RNA-binding proteins (RBPs). Number of proteins from the reference proteome (Uniprot) with chloroplastic, mitochondrial, nuclear and cytoplasmic localisation based on gene ontology (GO) annotations. Proteins from the reference proteome detected in our leaf RNA-binding proteome (RBPome) are coloured in red, whereas proteins not identified by plant RNA interactome capture (ptRIC) are coloured in grey.

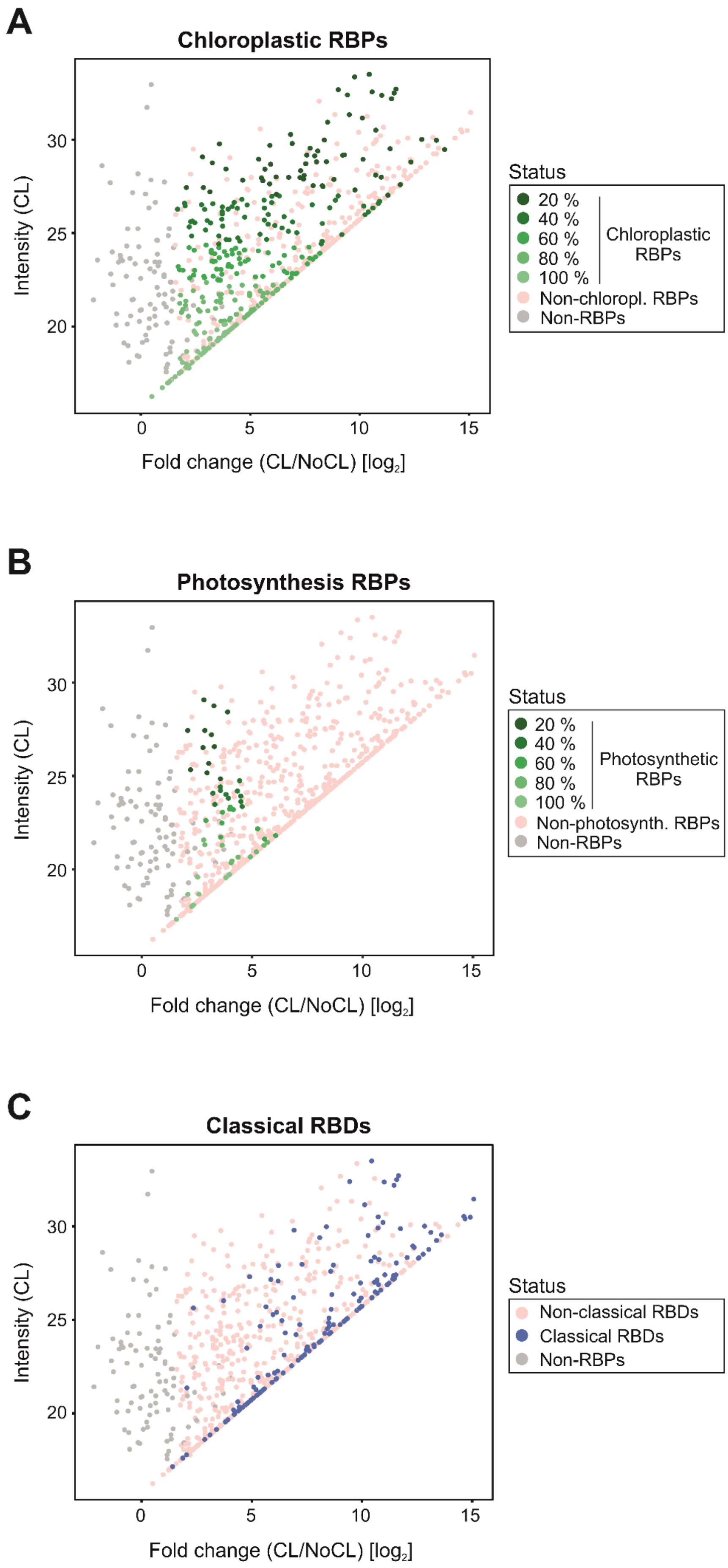

A
Chloroplastic RBPs
Intensity (CL)
Fold change (CL/NoCL) [log2]
Status
20 %
40 %
60 %
80 %
100 %
Chloroplastic RBPs
Non-chloropl. RBPs
Non-RBPs
B
Photosynthesis RBPs
Intensity (CL)
Fold change (CL/NoCL) [log2]
Status
20 %
40 %
60 %
80 %
100 %
Photosynthetic RBPs
Non-photosynth. RBPs
Non-RBPs
C
Classical RBDs
Intensity (CL)
Fold change (CL/NoCL) [log2]
Status
Non-classical RBDs
Classical RBDs
Non-RBPs

Figure S4. Chloroplastic and photosynthetic proteins are true RNA-binding proteins (RBPs). Scatter plots depict the log_2 fold change between no UV crosslinking (NoCL) and UV crosslinking (CL) treatments (log_2 FC[CL/NoCL]) and the signal intensity in the CL sample for each protein (dots) using data from four biological replicates. Proteins are coloured based on their chloroplastic localisation (**A**), role in photosynthesis (**B**) or presence of classical RNA-binding domains (RBDs) (**C**). For panels (**A**) and (**B**) the proteins were ranked based on their intensity in CL sample and coloured accordingly. Thus, the top 20% proteins with the highest intensity are coloured in dark green, the top 40% in lighter green, etc.

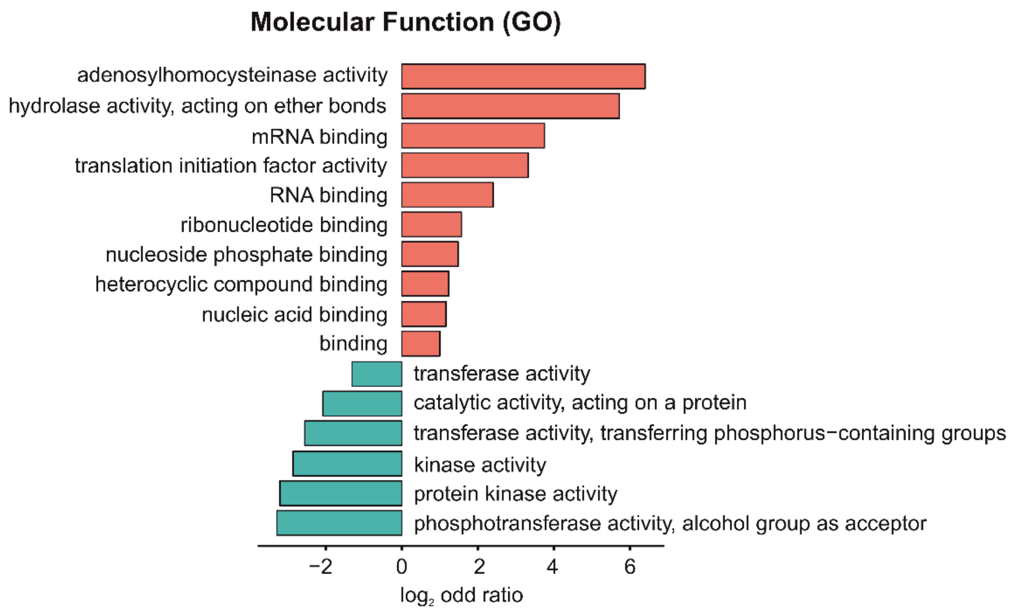

Figure S5. Annotation of RNA-binding proteins (RBPs) lacking known RNA-binding domains (RBDs). The most significantly enriched (red) or under-represented (blue) molecular function gene ontology (GO) terms of the leaf RBPs identified by plant RNA interactome capture (ptRIC) that do not contain known RBDs.

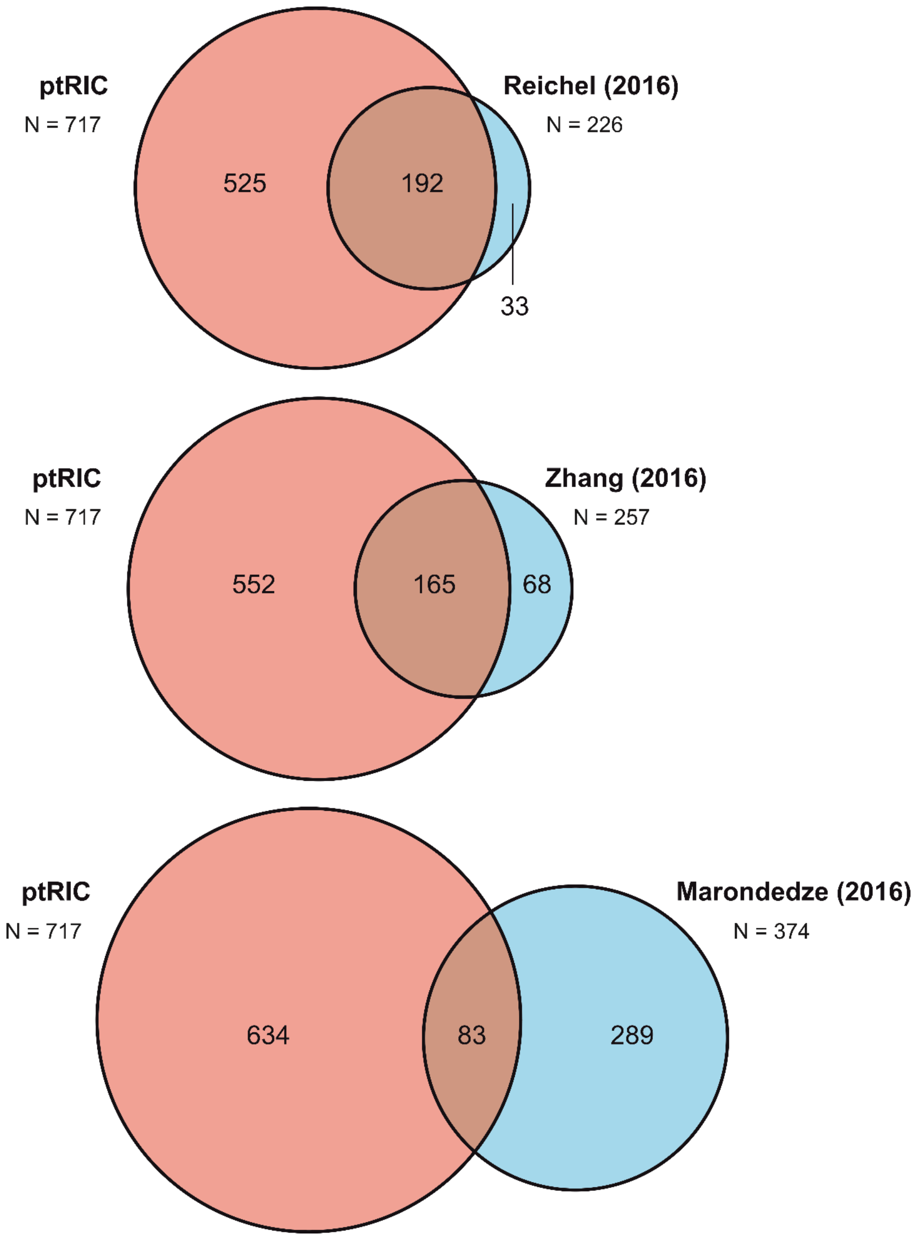

Figure S6. Overlap between the plant RNA interactome capture (ptRIC) leaf RNA-binding proteome (RBPome) and previous Arabidopsis RBPomes. Venn diagrams depict the overlap between the ptRIC leaf RBPome and each of the three previously published Arabidopsis RBPomes. The previous RIC studies used different plant tissues: Marondedze and colleagues used cell cultures and leaves, Zhang and colleagues used mesophyll protoplasts and Reichel and colleagues used etiolated seedlings.

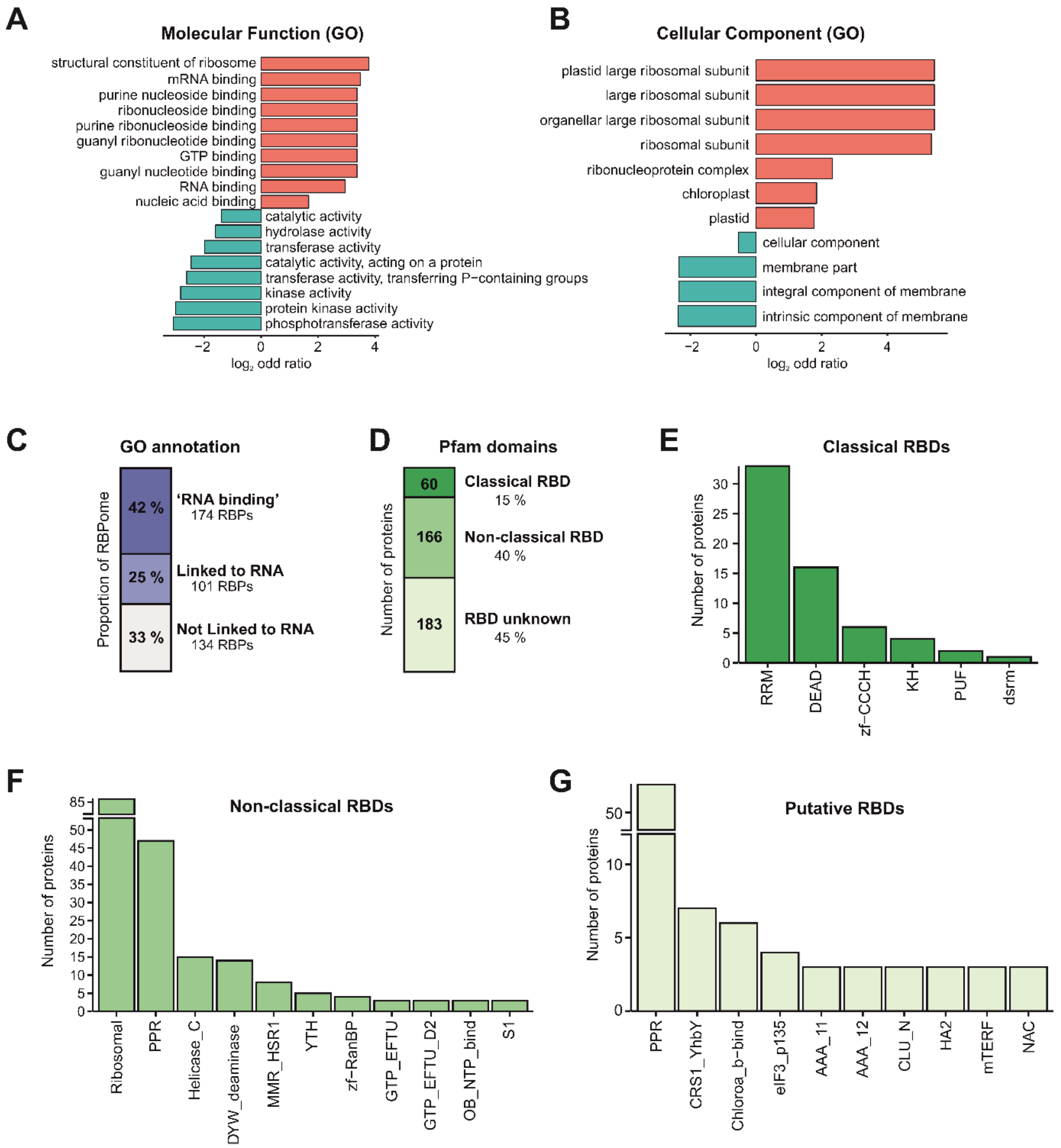

Figure S7. Insights into the plant RNA interactome capture (ptRIC)-specific leaf RNA-binding proteins (RBPs). Gene ontology (GO) analysis with ten of the most significantly enriched (red) or under-represented (blue) molecular function (**A**) or cellular component (**B**) GO terms of the ptRIC-specific leaf RNA-binding proteome (RBPome). (**C**) Proportion of the ptRIC-specific leaf RBPome with the GO annotation 'RNA binding', GO annotations 'linked to RNA biology' or with no GO annotations 'linked to RNA biology'. (**D**) Number of proteins harbouring classical, non-classical or no known RNA-binding domains (RBDs) in the ptRIC-specific leaf RBPome. (**E**) Number of proteins annotated as possessing classical RBDs, (**F**) non-classical RBDs or (**G**) putative RBDs in the ptRIC-specific leaf RBPome. For non-classical and putative RBD, only RBDs with at least three protein counts are shown.
