# Supplementary material for: Discovering the RNA-Binding Proteome of Plant Leaves with an Improved RNA Interactome Capture Method
Source: Biomolecules. 2020 Apr 24;10(4):661. doi: 10.3390/biom10040661 (PMC7226388; doi:10.3390/biom10040661)
Supplement: Supplementary file 1 [file biomolecules-10-00661-s001.zip › biomolecules-776768-supplementary-final version/Supplementary_materials_methods.pdf]

Supplementary materials and methods

1. Sample preparation for mass spectrometry

Samples were prepared for mass spectrometry using the standard FASP (filter aided sample preparation) method with minor modifications [1]. Briefly, proteins were mixed with urea buffer (8 M urea (Sigma-Aldrich cat. no. U1250), 100 mM TEAB (triethylammonium bicarbonate buffer; Sigma-Aldrich cat. no. T7408)), loaded in a 10 KDa cut-off Vivacon filter unit (Sartorius; cat. no. VN01H01) and centrifuged at 15,000g for 30 minutes at 20 °C. Proteins were washed six times with 200 µL urea buffer to remove any detergents from the sample. Protein reduction was performed with 200 µL urea buffer supplemented with 10 mM TCEP (Tris-(2-Carboxyethyl)phosphine); Sigma-Aldrich cat. no. C4706) for 30 minutes at room temperature. Protein alkylation was performed by adding CA (Chloracetamide; Sigma-Aldrich cat. no. C0267) to a final concentration of 50 mM for 30 minutes, at room temperature and in the dark. The samples were washed three times with 200 µL of a buffer containing 6 M urea and 50 mM TEAB and centrifuged (15,000g, 30 minutes, 20 °C). Proteins were digested inside the filter unit by adding 1 µg of Lysyl endopeptidase (Wako, cat. no. 125-05061) in 99 µL of a buffer containing 1 M urea and 50 mM TEAB for 4 hours at 37 °C. Polypeptides were further digested with 1 µg of trypsin (Promega, cat. no. V5111) in 99 µL of a buffer containing 50 mM TEAB overnight at 37°C. The digested peptides were harvested by centrifugation (15,000g, 25 minutes, 20 °C). To increase the peptide recovery, the filters were washed once with 0.1 % TFA (Trifluoroacetic acid; Thermo Fisher Scientific, cat. no. 85183), followed by one wash with 50 % acetonitrile (ACN; Honeywell, cat. no. 14261) and 0.1 % TFA. Peptides were harvested by centrifugation (15,000g, 25 minutes, 20 °C) and eluates were dried using a SpeedVac (Thermo Fisher Scientific, cat. no. SPD210-115). The acidified tryptic digests were desalted on home-made 2 disc C18 StageTips as described [2]. After elution from the StageTips, samples were dried using a vacuum concentrator (Eppendorf) and the peptides were taken up in 10 µL 0.1 % formic acid solution (FA; Fisher Scientific, cat. No. 10596814).

2. Mass spectrometry analyses

Experiments were performed on an Orbitrap Elite instrument (Thermo Fisher Scientific) [3] that was coupled to an EASY-nLC 1000 liquid chromatography (LC) system

(Thermo Fisher Scientific). The LC was operated in the one-column mode. The analytical column was a fused silica capillary (75 µm × 35 or 50 cm) with an integrated PicoFrit emitter (New Objective) packed in-house with Reprosil-Pur 120 C18-AQ 1.9 µm resin (Dr. Maisch). The analytical column was encased by a column oven (Sonation) and attached to a nanospray flex ion source (Thermo Fisher Scientific). The column oven temperature was adjusted to 45 °C during data acquisition. The LC was equipped with two mobile phases: solvent A (0.1% FA in water) and solvent B (0.1% FA in ACN). All solvents were of UPLC grade (Sigma-Aldrich). Peptides were directly loaded onto the analytical column with a maximum flow rate that would not exceed the set pressure limit of 980 bar (usually around 0.6 – 1.0 µL/minute). Peptides were subsequently separated on the analytical column by running a 140 minute gradient of solvent A and solvent B (start with 7% B; gradient 7% to 35% B for 120 minutes; gradient 35% to 100% B for 10 minutes and 100% B for 10 minutes) at a flow rate of 300 nL/minute. The mass spectrometer was operated using Xcalibur software (version 2.2 SP1.48). The mass spectrometer was set in the positive ion mode. Precursor ion scanning was performed in the Orbitrap analyser (FTMS; Fourier Transform Mass Spectrometry) in the scan range of m/z 300-1800 and at a resolution of 60000 with the internal lock mass option turned on (lock mass was 445.120025 m/z, polysiloxane) [4]. Product ion spectra were recorded in a data dependent fashion in the ion trap (ITMS) in a variable scan range and at a rapid scan rate. The ionization potential (spray voltage) was set to 1.8 kV. Peptides were analysed using a repeating cycle consisting of a full precursor ion scan (3.0×10^6 ions or 50 ms) followed by 15 product ion scans (1.0×10^4 ions or 50 ms) where peptides are isolated based on their intensity in the full survey scan (threshold of 500 counts) for tandem mass spectrum (MS2) generation that permits peptide sequencing and identification. Collision induced dissociation (CID) energy was set to 35% for the generation of MS2 spectra. During MS2 data acquisition dynamic ion exclusion was set to 120 seconds with a maximum list of excluded ions consisting of 500 members and a repeat count of one. Ion injection time prediction, preview mode for the FTMS, monoisotopic precursor selection and charge state screening were enabled. Only charge states higher than 1 were considered for fragmentation.

RAW spectra were submitted to an Andromeda [5] search in MaxQuant (1.5.3.30) using the default settings [6]. Label-free quantification and match-between-runs was

activated [7]. We downloaded the reference *Arabidopsis thaliana* database from NCBI (Thale cress, ARA_GCF_000001735.4_TAIR10.1_protein.fasta, downloaded 20/02/2019). All searches included a contaminants database search (as implemented in MaxQuant) to estimate the level of contamination with known MS contaminants. Andromeda searches allowed oxidation of methionine residues (16 Da) and acetylation of the protein N-terminus (42 Da) as dynamic modifications and the static modification of cysteine (57 Da, alkylation with iodoacetamide). Enzyme specificity was set to "Trypsin/P" with two missed cleavages allowed. The instrument type in Andromeda searches was set to Orbitrap and the precursor mass tolerance was set to ±20 ppm (first search) and ±4.5 ppm (main search). The MS/MS match tolerance was set to ±0.5 Da. The peptide spectrum match FDR and the protein FDR were set to 0.01 (based on target-decoy approach). Minimum peptide length was 7 amino acids. For protein quantification unique and razor peptides were allowed. Modified peptides were allowed for quantification. The minimum score for modified peptides was 40. Label-free protein quantification was switched on, and unique and razor peptides were considered for quantification with a minimum ratio count of 2. Retention times were recalibrated based on the built-in nonlinear time-rescaling algorithm. MS/MS identifications were transferred between LC-MS/MS runs with the "match between runs" option in which the maximal match time window was set to 0.7 minutes and the alignment time window set to 20 minutes. The quantification is based on the "value at maximum" of the extracted ion current. At least two quantitation events were required for a quantifiable protein.

3. Bioinformatic analyses of the leaf RBPome

The reference Arabidopsis proteome was downloaded (July 2019) from Uniprot (www.uniprot.org) and was used for gene ontology (GO) and Pfam annotations. A superset of Arabidopsis RNA-binding proteins (RBPs) was generated by adding the leaf RBPs identified by plant RNA interactome capture (ptRIC) to the Arabidopsis RBPs identified in previous studies [8–10] and standardised by Hentze and colleagues [11].

Gene set enrichment analyses were performed by comparing frequencies of GO terms in the leaf RNA-binding proteome (RBPome) with the frequency of the same GO terms in either the reference Arabidopsis proteome or the superset of Arabidopsis RBPs. Statistical testing was performed applying Fisher's exact test and Bonferroni corrected

p-values were used to account for multiple testing. We allowed for propagation of GO terms to account for the intrinsic hierarchy of GO terms. All statistical testing was performed using GOATOOLS [12] with the basic GO file version 1.2 (released on 01.07.2019). Significantly enriched GO terms were divided into the different GO categories ’cellular component and ’molecular function’. For each category, $\log_2$ odds ratios of some of the most significantly enriched GO terms were calculated. The occurrence of significantly enriched GO terms that were absent in the RBPome was artificially set to the lowest biologically sensible value (1) to enable calculation of $\log_2$ odds ratios. The links to RNA biology of the identified RBPs were determined as described previously [13,14].

The Pfam domains were categorised into classical RNA-binding domains (RBDs), non-classical RBDs and domains not known to be linked to RNA-binding (putative RBDs) by manual annotation based on data from previous studies [13,15] as previously described [13]. Two analyses were performed. First, hierarchical analysis of Pfam distribution whereby proteins were analysed to determine whether they contained a Pfam code that corresponded to a classical, non-classical or putative RBD (RBD unknown). Classical was dominant over non-classical and non-classical was dominant over unknown. If one protein was associated with more than one RBD, the most dominant category was selected for the protein. Second, the distribution of Pfam names in each of the three groups (classical, non-classical and putative) was analysed. The following Pfam names were merged into groups: within classical RBDs, all Pfam names beginning with ‘RRM’, ’KH’ and ‘zf-CCCH’ were merged; within non-classical RBDs, all Pfam names beginning with ’Ribosomal’ were merged; and within putative RBDs, all Pfam names beginning with ’PPR’ were merged.

To analyse the overlap between the different Arabidopsis RNA interactome captures (RICs), the Arabidopsis RBPomes were obtained from the uniformed dataset published by Hentze and colleagues [11]. The majority of the graphs were generated using the ggplot2 package within R [16]. The Venn diagrams were generated using the VennDiagram package within R [17] and the heat map was generated using the ggdendro and grid packages within R [18].

References

1. Wiśniewski, J.R.; Zougman, A.; Nagaraj, N.; Mann, M. Universal sample preparation method for proteome analysis. *Nat. Methods* **2009**, *6*, 359–62.
2. Rappsilber, J.; Mann, M.; Ishihama, Y. Protocol for micro-purification, enrichment, pre-fractionation and storage of peptides for proteomics using StageTips. *Nat. Protoc.* **2007**, *2*, 1896–1906.
3. Michalski, A.; Damoc, E.; Lange, O.; Denisov, E.; Nolting, D.; Müller, M.; Viner, R.; Schwartz, J.; Remes, P.; Belford, M.; et al. Ultra high resolution linear ion trap orbitrap mass spectrometer (orbitrap elite) facilitates top down LC MS/MS and versatile peptide fragmentation modes. *Mol. Cell. Proteomics* **2012**, *11*, 1–11.
4. Olsen, J. V.; de Godoy, L.M.F.; Li, G.; Macek, B.; Mortensen, P.; Pesch, R.; Makarov, A.; Lange, O.; Horning, S.; Mann, M. Parts per million mass accuracy on an orbitrap mass spectrometer via lock mass injection into a C-trap. *Mol. Cell. Proteomics* **2005**, *4*, 2010–2021.
5. Cox, J.; Neuhauser, N.; Michalski, A.; Scheltema, R.A.; Olsen, J. V.; Mann, M. Andromeda: a peptide search engine integrated into the MaxQuant environment. *J. Proteome Res.* **2011**, *10*, 1794–1805.
6. Cox, J.; Mann, M. MaxQuant enables high peptide identification rates, individualized p.p.b.-range mass accuracies and proteome-wide protein quantification. *Nat. Biotechnol.* **2008**, *26*, 1367–1372.
7. Cox, J.; Hein, M.Y.; Luber, C.A.; Paron, I.; Nagaraj, N.; Mann, M. Accurate proteome-wide label-free quantification by delayed normalization and maximal peptide ratio extraction, termed MaxLFQ. *Mol. Cell. Proteomics* **2014**, *13*, 2513–2526.
8. Reichel, M.; Liao, Y.; Rettel, M.; Ragan, C.; Evers, M.; Alleaume, A.-M.; Horos, R.; Hentze, M.W.; Preiss, T.; Millar, A.A. In planta determination of the mRNA-binding proteome of Arabidopsis etiolated seedlings. *Plant Cell* **2016**, *28*, 2435–2452.
9. Zhang, Z.; Boonen, K.; Ferrari, P.; Schoofs, L.; Janssens, E.; van Noort, V.; Rolland, F.; Geuten, K. UV crosslinked mRNA-binding proteins captured from leaf mesophyll protoplasts. *Plant Methods* **2016**, *12*, 42.
10. Marondedze, C.; Thomas, L.; Serrano, N.L.; Lilley, K.S.; Gehring, C. The RNA-binding protein repertoire of *Arabidopsis thaliana*. *Sci. Rep.* **2016**, *6*, 29766.
11. Hentze, M.W.; Castello, A.; Schwarzl, T.; Preiss, T. A brave new world of RNA-binding proteins. *Nat. Rev. Mol. Cell Biol.* **2018**, *19*, 327–341.
12. Klopfenstein, D. V.; Zhang, L.; Pedersen, B.S.; Ramírez, F.; Vesztrocy, A.W.; Naldi, A.; Mungall, C.J.; Yunes, J.M.; Botvinnik, O.; Weigel, M.; et al. GOATOOLS: A Python library for Gene Ontology analyses. *Sci. Rep.* **2018**, *8*, 1–17.
13. Castello, A.; Fischer, B.; Eichelbaum, K.; Horos, R.; Beckmann, B.M.; Strein, C.; Davey, N.E.; Humphreys, D.T.; Preiss, T.; Steinmetz, L.M.; et al. Insights into RNA biology from an atlas of mammalian mRNA-binding proteins. *Cell* **2012**, *149*, 1393–1406.
14. Beckmann, B.M.; Horos, R.; Fischer, B.; Castello, A.; Eichelbaum, K.; Alleaume, A.-M.; Schwarzl, T.; Curk, T.; Foehr, S.; Huber, W.; et al. The RNA-binding proteomes from yeast to man harbour conserved enigmRBPs. *Nat. Commun.* **2015**, *6*, 10127.

15. Castello, A.; Fischer, B.; Frese, C.K.; Horos, R.; Alleaume, A.M.; Foehr, S.; Curk, T.; Krijgsveld, J.; Hentze, M.W. Comprehensive identification of RNA-binding domains in human cells. *Mol. Cell* **2016**, *63*, 696–710.
16. Wickham, H. *ggplot2: Elegant Graphics for Data Analysis*; Springer-Verlag: New York, 2016; ISBN 978-3-319-24277-4.
17. Chen, H.; Boutros, P.C. VennDiagram: a package for the generation of highly-customizable Venn and Euler diagrams in R. *BMC Bioinformatics* **2011**, *12*, 35.
18. R Core Team. *R: A Language and Environment for Statistical Computing*; R Foundation for Statistical Computing: Viena, Austria, 2017.
